# Biological and Chemical Adaptation to Endogenous Hydrogen Peroxide Production in *Streptococcus pneumoniae* D39

**DOI:** 10.1128/mSphere.00291-16

**Published:** 2017-01-04

**Authors:** John P. Lisher, Ho-Ching Tiffany Tsui, Smirla Ramos-Montañez, Kristy L. Hentchel, Julia E. Martin, Jonathan C. Trinidad, Malcolm E. Winkler, David P. Giedroc

**Affiliations:** a Department of Chemistry, Indiana University, Bloomington, Indiana, USA; b Graduate Program in Biochemistry, Indiana University, Bloomington, Indiana, USA; c Department of Biology, Indiana University, Bloomington, Indiana, USA; d Department of Chemistry, Laboratory for Biological Mass Spectrometry, Indiana University, Bloomington, Indiana, USA; e Department of Molecular and Cellular Biochemistry, Indiana University, Bloomington, Indiana, USA; University of Iowa

**Keywords:** *Streptococcus pneumoniae*, hydrogen peroxide stress, pyruvate oxidase, sulfenylation

## Abstract

The catalase-negative, facultative anaerobe *Streptococcus pneumoniae* D39 is naturally resistant to hydrogen peroxide (H_2_O_2_) produced endogenously by pyruvate oxidase (SpxB). Here, we investigate the adaptive response to endogenously produced H_2_O_2_. We show that lactate oxidase, which converts lactate to pyruvate, positively impacts pyruvate flux through SpxB and that *ΔlctO* mutants produce significantly lower H_2_O_2_. In addition, both the SpxB pathway and a candidate pyruvate dehydrogenase complex (PDHC) pathway contribute to acetyl coenzyme A (acetyl-CoA) production during aerobic growth, and the pyruvate format lyase (PFL) pathway is the major acetyl-CoA pathway during anaerobic growth. Microarray analysis of the D39 strain cultured under aerobic versus strict anaerobic conditions shows upregulation of *spxB*, a gene encoding a rhodanese-like protein (locus tag *spd0091*), *tpxD*, *sodA*, *piuB*, *piuD*, and an Fe-S protein biogenesis operon under H_2_O_2_-producing conditions. Proteome profiling of H_2_O_2_-induced sulfenylation reveals that sulfenylation levels correlate with cellular H_2_O_2_ production, with endogenous sulfenylation of ≈50 proteins. Deletion *of tpxD* increases cellular sulfenylation 5-fold and has an inhibitory effect on ATP generation. Two major targets of protein sulfenylation are glyceraldehyde-3-phosphate dehydrogenase (GapA) and SpxB itself, but targets also include pyruvate kinase, LctO, AdhE, and acetate kinase (AckA). Sulfenylation of GapA is inhibitory, while the effect on SpxB activity is negligible. Strikingly, four enzymes of capsular polysaccharide biosynthesis are sulfenylated, as are enzymes associated with nucleotide biosynthesis via ribulose-5-phosphate. We propose that LctO/SpxB-generated H_2_O_2_ functions as a signaling molecule to downregulate capsule production and drive altered flux through sugar utilization pathways.

**IMPORTANCE** Adaptation to endogenous oxidative stress is an integral aspect of *Streptococcus pneumoniae* colonization and virulence. In this work, we identify key transcriptomic and proteomic features of the pneumococcal endogenous oxidative stress response. The thiol peroxidase TpxD plays a critical role in adaptation to endogenous H_2_O_2_ and serves to limit protein sulfenylation of glycolytic, capsule, and nucleotide biosynthesis enzymes in *S. pneumoniae*.

## Abstract (precis)

Adaptation to endogenous oxidative stress is an integral aspect of *Streptococcus pneumoniae* colonization and virulence. In this work, we identify key transcriptomic and proteomic features of the pneumococcal endogenous oxidative stress response. The thiol peroxidase TpxD plays a critical role in adaptation to endogenous H_2_O_2_ and serves to limit protein sulfenylation of glycolytic, capsule, and nucleotide biosynthesis enzymes in *S. pneumoniae*.

## INTRODUCTION

**FIG 1:**
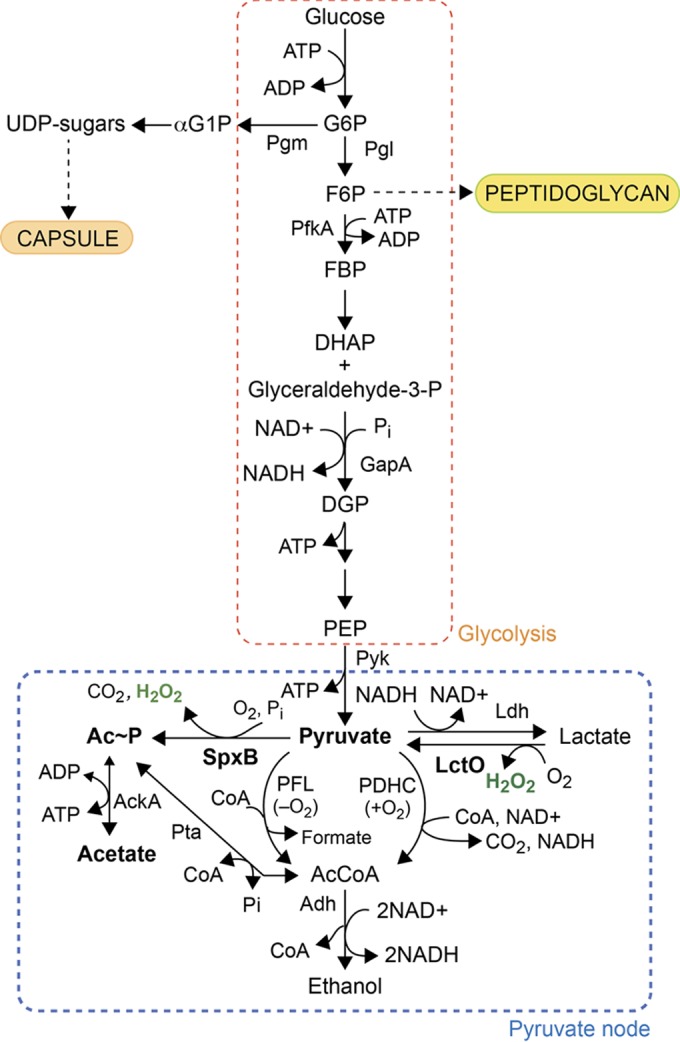
Schematic overview of glycolysis and the fates of pyruvate in *Streptococcus pneumoniae* D39 (serotype 2). In this study, we provide genetic evidence that the pyruvate dehydrogenase complex (PDHC) is a functional pathway for the production of acetyl-CoA under aerobic conditions, consistent with previous findings in the TIGR4 strain (41), and that pyruvate formate lyase (PFL) is the major pathway for acetyl-CoA synthesis under anaerobic conditions. Note that acetate kinase (AckA) and phosphate acetyltransferase (Pta) are reversible enzymes and are represented here with double-headed arrows (8).

*Streptococcus pneumoniae* (pneumococcus) is a Gram-positive facultative anaerobe that is the causative agent of significant respiratory and invasive disease, including sinusitis, otitis media, pneumonia, and meningitis, which annually lead to significant morbidity and mortality worldwide (1). Within the human host, pneumococcus is exposed to conditions of variable oxygen levels depending on the site of colonization or infection, from 20% oxygen (air) on the airway surface on top of the nasopharyngeal mucus layer, to ~5% in the lower respiratory tract, to virtually anaerobic conditions in the blood (2). As a lactic acid bacterium, *S. pneumoniae* is characterized by a fermentative metabolism lacking both the respiratory electron transport chain and the tricarboxylic acid cycle (3–5). Pyruvate, the end product of glycolysis, is used as a precursor to make acetyl coenzyme A (acetyl-CoA) and acetyl-phosphate (and ultimately ATP), while l-lactate is used to regenerate NAD^+^ via lactate dehydrogenase (Ldh) (Fig. 1) (6). In the presence of molecular oxygen, lactate oxidase (LctO) converts l-lactate to pyruvate and hydrogen peroxide (H_2_O_2_). Pyruvate oxidase (SpxB) then catalyzes the conversion of pyruvate to the phosphoryl donor, acetyl phosphate (Ac~P), releasing CO_2_ and H_2_O_2_ (Fig. 1).

SpxB is considered the major contributor of H_2_O_2_ and Ac~P production as Δ*spxB* mutants produce only 10% and 18% of the amounts of H_2_O_2_ and Ac~P, respectively, compared to the *spxB*^+^ parent (7, 8). Ac~P is converted to acetate by acetate kinase (AckA) generating one equivalent of ATP from ADP (8) (Fig. 1). Ac~P is also a precursor to acetyl-CoA, which is essential for the synthesis of fatty acid intermediates. Two other likely pneumococcal enzymes that synthesize acetyl-CoA include a pyruvate dehydrogenase complex (PDHC) and pyruvate formate lyase (PFL) (Fig. 1). It is unclear if *S. pneumoniae* possesses an active pyruvate dehydrogenase under aerobic growth conditions (9, 10). Pyruvate formate lyase, whose activity is oxygen sensitive (11), likely functions under anaerobic conditions. However, the contributions of these three pathways under aerobiosis or anaerobiosis have not been examined.

Recent studies firmly connect the pyruvate node of central metabolism through SpxB to capsule production and cell wall status, which are strong virulence determinants required for invasive disease (12–16). SpxB may play different roles in various aspects of virulence, depending on the serotype. *spxB* knockout mutants of strain D39 (serotype 2) has been shown to be attenuated in an intranasal murine model (12, 15, 17), although hypervirulent colonies of *S. pneumoniae* serotype 1 contain mutations in *spxB* (18). *S. pneumoniae* can endogenously generate up to of 2 mM hydrogen peroxide (H_2_O_2_) aerobically (7) under laboratory conditions. This production of H_2_O_2_ highlights an interesting interplay between the beneficial fitness advantages afforded by SpxB and its detrimental effect on evading human macrophages (18). Endogenous H_2_O_2_ from *S. pneumoniae* is likely used to kill other competing microbes in the community (13), while pneumococcus is naturally resistant to H_2_O_2_, thus providing the bacterium an advantage during colonization of the upper respiratory tract (7, 19).

Free reduced iron [Fe(II)] is a major contributor to reactive oxygen species (ROS) via the catalytic generation of the highly reactive hydroxyl radical, OH·, which damages biomolecules (20). Bioavailable or non-protein-associated Fe(II) levels are ~0.2 mM under aerobic conditions as measured by electron paramagnetic resonance spectroscopy (7). Interestingly, *S. pneumoniae* is capable of tolerating high levels of bioavailable Fe and H_2_O_2_, despite the formation of hydroxyl radicals during aerobic growth (7). These data suggest that *S. pneumoniae* possesses a robust oxidative repair mechanism or mechanisms. In most bacteria, dedicated transcriptional regulators, OxyR or PerR, sense H_2_O_2_ stress and activate transcription of genes encoding enzymes involved in H_2_O_2_ detoxification and repair (21, 22). However, *S. pneumoniae* does not encode these regulons; instead, other known or candidate transcriptional regulators, including SpxR (15), Rgg (23), RitR (24), NmlR (25), PsaR (26), and CiaRH (27) have been linked to gene regulation, either directly or indirectly, in response to oxidative stress (2). This suggests that the oxidative stress response of *S. pneumoniae* may be integrated into other regulatory networks, a finding consistent with a recent microarray study designed to understand how the unencapsulated laboratory *S. pneumoniae* R6 strain adapts to oxygen (23).

In mammalian cells, it is well established that reversible thiol oxidation plays an important role in the signal transduction (28). Cysteine thiols can accommodate a range of distinct chemical derivatizations, ranging from *S*-oxidation to create sulfenic (-SOH), sulfinic (-SO_2_H), and sulfonic (-SO_3_H) acid moieties, *S*-nitrosylation (-SNO), *S*-sulfhydration or persulfidation (-SSH), *S*-alkylation via Michael addition to electrophilic carbon centers (-SCH_2_OR), and formation of mixed disulfides with cellular thiols, e.g., *S*-glutathionylation or *S*-bacillithionylation (29). The reversible *S*-hydroxylation (sulfenylation) of thiols by H_2_O_2_ is of particular interest, since exogenous H_2_O_2_ is part of the oxidative burst induced by neutrophils. While there are many studies of proteomic profiling of thiol-specific modifications reported in eukaryotic systems (for reviews, see references 30 and 31), there are comparatively fewer studies carried out in bacteria (32). One report mapped oxidation-sensitive cysteines in two bacterial pathogens, *Pseudomonas aeruginosa* and *Staphylococcus aureus*, by measuring differential degrees of thiol modifications in the presence of a short burst of 10 mM exogenous H_2_O_2_ (32) as a model for exogenous oxidative stress that might be encountered in transitioning from a commensal to virulent lifestyle (29). Over 200 proteins were found to contain H_2_O_2_-sensitve cysteines measured indirectly by a loss of a free thiol in the proteome; however, the nature of the modification(s) could not be determined using this approach (32).

Low-molecular-weight (LMW) thiols, including glutathione and l-cysteine, may function to protect protein thiols via *S*-thiolation in *S. pneumoniae*. Glutathione is known to play an important role in metal homeostasis and combating the effects of redox-cycling molecules, including paraquat (33, 34). Although *S. pneumoniae* is incapable of synthesizing glutathione, it can readily import exogenous glutathione via the GshT glutathione transporter (33). Thiol-dependent repair systems, including thiol peroxidase (TpxD), glutathione peroxidase (Gpx), and the thioredoxin (TrxA)-thioredoxin reductase (TrxB) pair, play a significant role in the repair of oxidized thiols (30, 35, 36).

In this study, we employ genetic approaches to identify pneumococcal proteins in addition to SpxB that are involved in the pyruvate to Ac~P and H_2_O_2_ production pathways. We provide evidence that LctO contributes to the production of H_2_O_2_ via increased pyruvate flux through SpxB and also directly through its H_2_O_2_ production activity. We provide genetic support for a functional pyruvate dehydrogenase complex by showing that Δ*spxB* ΔPDHC mutations are synthetically lethal under aerobic growth conditions. To investigate how a virulent *S. pneumoniae* strain adapts to its own persistent production of H_2_O_2_ by SpxB and LctO, we performed a microarray analysis comparing differential gene expression under aerobic or anaerobic conditions. Profiling of proteome sulfenylation indicates that relative levels of sulfenylation correlate with relative hydrogen peroxide concentrations. We show that TpxD and LMW thiols play a major role in the control and repair, respectively, of proteome sulfenylation, a major posttranslational modification that results from H_2_O_2_ stress (37). Approximately 50 cytoplasmic proteins are sulfenylated in cells, with major targets the glycolytic enzyme glyceraldehyde-3-phosphate dehydrogenase (GapA) and SpxB itself. We propose that pneumococcus deploys a chemical adaptation strategy superimposed on transcriptomic changes in response to endogenous H_2_O_2_ to regulate a number of key cellular processes, including biosynthesis of the capsule.

## RESULTS

### Lactate oxidase (LctO) contributes to endogenous H_2_O_2_ levels.

**FIG 2:**
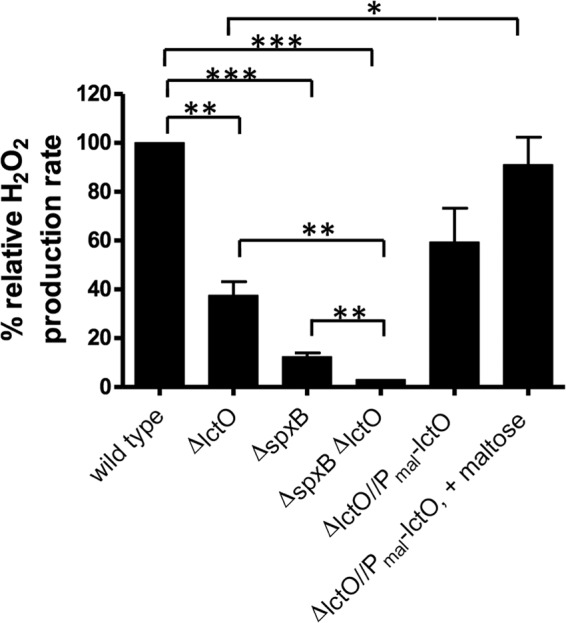
Rates of H_2_O_2_ production in the parent and mutant strains used in this study. Rates of H_2_O_2_ production normalized to culture densities were determined relative to that produced by parent strain D39 (IU1690) in BHI broth as described in Materials and Methods. The strains used were the D39 WT (IU1690), *ΔlctO* (IU2633), *ΔspxB* (IU2181), and *ΔspxB ΔlctO* (IU3284) mutants, and *lctO* complemented strain (*ΔlctO*//P_mal_-*lctO* [IU2952]) in the absence or presence of 1% inducer maltose. Full genotypes of the strains are listed in Table S1A. Biological replicates were performed three or more times, and standard errors of the mean are shown. *, *P* < 0.05, **, *P* < 0.01, and ***, *P* < 0.001, by 2-tailed unpaired *t* test.

In a transposon insertion screen (15) designed to identify possible regulators of *spxB* and other genes that impact H_2_O_2_ production in *S. pneumoniae* strain R6, we identified a transposon insertion mutant that appeared more opaque than the R6 parent. Since colony opaqueness often correlates with a lower H_2_O_2_ production rate (15, 19), we measured this and found that this mutant generates H_2_O_2_ at a rate ~40% relative to that of the wild-type R6 strain (data not shown). Using inverse PCR, we mapped the insertion site to the lactate oxidase gene (*lctO*) and determined that the insertion resulted in truncating the LctO at I152, thereby inactivating the enzyme. A backcross of this insertion into the D39 strain similarly reduces H_2_O_2_ production to 40% relative to the D39 parent strain (Fig. 2). A complemented strain containing *lctO* in the neutral CEP site under control of the maltose promoter (P_mal_) (38) grown in the presence of 1% maltose produces levels of H_2_O_2_ similar to those of the parent strain, demonstrating that LctO is involved in H_2_O_2_ production (Fig. 2).

SpxB was identified as the major producer of endogenous H_2_O_2_, because Δ*spxB* strains produce less than 13% of the total H_2_O_2_ (Fig. 2) (7, 15) produced by the *spxB*^+^ parent. It is therefore worth noting that a single *lctO* deletion also results in a significant 62% decrease in H_2_O_2_ production relative to the wild-type parent. Since LctO catalyzes the conversion of O_2_ and lactate to H_2_O_2_ and pyruvate, which is a substrate for SpxB, the large reduction in H_2_O_2_ in the Δ*lctO* mutant can be explained by its inability to recycle lactate into pyruvate and hence a decreased flux of pyruvate into the SpxB-mediated reaction (Fig. 1). In addition, the amount of H_2_O_2_ production obtained from the double deletion mutant (3%) is statistically smaller than that obtained from the single Δ*spxB* (13%) or Δ*lctO* (38%) mutants (Fig. 2), indicating that LctO also directly contributes to the production of H_2_O_2_.

### A deletion of *lctO* affects sensitivity to exogenous H_2_O_2_ to a similar extent as in a *ΔspxB* mutant.

Previous studies showed somewhat paradoxically that deletion of *spxB* increases sensitivity to exogenously added H_2_O_2_ (20 mM) (7). The origin of this phenotype is unclear since endogenous H_2_O_2_ production is significantly lower in this mutant (Fig. 2). Pneumococci lacking *lctO* are equally highly sensitive to 20 mM exogenous H_2_O_2_, as is the Δ*spxB* Δ*lctO* mutant relative to the Δ*spxB* strain (see Table S1C in the supplemental material). The degree of H_2_O_2_ sensitivity is far more pronounced in these strains than in other strains harboring deletions in genes that potentially protect cells from ROS, including *sodA* and *tpxD* (data not shown) (39). This suggests a physiological distinction between endogenous and exogenous sources of H_2_O_2_ production, further elucidated here.

10.1128/mSphere.00291-16.1 TABLE S1  Strains, DNA primers, hydrogen peroxide sensitivity of selected pneumococcal strains, and microarray analysis of genes that change expression upon a switch from anaerobic to aerobic conditions. (A) Strains of *Streptococcus pneumoniae* used in this study. (B) Oligonucleotide primers used in this study (order follows Table S1A). (C) H_2_O_2_ sensitivity of various *S. pneumoniae* D39 strains. (D) Changes in relative transcript amounts in *Streptococcus pneumoniae* D39 grown exponentially in BHI broth with limited aeration (aerobic growth) compared with growth under anaerobic condition. + or −, upregulated (+) or downregulated (−) under aerobic conditions. Download Table S1, XLS file, 0.1 MB. Copyright © 2017 Lisher et al. 2017 Lisher et al. This content is distributed under the terms of the Creative Commons Attribution 4.0 International license.

### A candidate pyruvate dehydrogenase complex provides a functional pathway for the production of acetyl-CoA.

In addition to H_2_O_2_ production, SpxB also contributes to the production of a majority, but not all, of intracellular acetyl phosphate, as indicated by the 87% reduction of intracellular acetyl phosphate (Ac~P) in a Δ*spxB* mutant compared with the *spxB*^+^ parent (8). Ac~P is a precursor to acetyl-CoA, which is essential for the synthesis of fatty acid intermediates (Fig. 1). The two other pneumococcal enzymes that synthesize Ac~P comprise the phosphotransacetylase (Pta)-AckA pathway (8), which is downstream from a pyruvate dehydrogenase complex (PDHC) pathway (Fig. 1). It is controversial whether *S. pneumoniae* possesses an active pyruvate dehydrogenase under aerobic growth conditions (9). Four genes of the D39 genome (locus tags *spd1025* to *spd1028*) have similarity to an acetoin or a pyruvate dehydrogenase complex gene but were reported to have none of the predicted functions in a previous report (10). To determine if this complex is an acetoin dehydrogenase complex, we conducted a Vogues-Proskauer (VP) test and found no acetoin production in *S. pneumoniae* under laboratory growth conditions in brain heart infusion (BHI) (data not shown). Furthermore, *S. pneumoniae* D39 encodes only one protein, AcuB, annotated as an “acetoin utilization protein” whose function is unknown, and lacks other acetoin metabolic enzymes—e.g., acetoin reductase (40). A bioinformatics search of the pneumococcal genome (R6 and D39) (4, 5) further reveals that the pathway for acetoin production is not complete.

These findings suggest that *spd1025* to *spd1028* may well encode a bona fide pyruvate dehydrogenase complex. In strong support of this assignment, deletion of *S. pneumoniae* TIGR4 genes *sp1163* and *sp1164* (corresponding to *spd1127* and *spd1128* in the D39 strain) results in significantly reduced (≈50%) acetyl-CoA levels (Fig. 1) (41). We show here that *spd1025* to *spd1028* are absolutely essential for growth in a Δ*spxB* strain. We can readily construct mutants containing a deletion of these four genes encoding the putative PDHC, therefore demonstrating that they not essential for growth in a wild-type background. However, it is not possible to construct a double Δ*spxB* ΔPDHC mutant, showing that Δ*spxB* and ΔPDHC mutations are synthetically lethal (Table 1). Transformation of a ΔPDHC (Δ*spd1025*–*spd1028*) amplicon into Δ*spxB* strains or a Δ*spxB* amplicon into ΔPDHC strains yielded no colonies, while transformation of Δ*spxB* or ΔPDHC amplicons into wild-type strains and transformation of positive control Δ*pbp1b* amplicons into ΔPDHC or Δ*spxB* strains yielded many colonies. These results suggest that under aerobic conditions, either the SpxB or PDHC pathway must be present to produce acetyl-CoA, either directly from pyruvate by this candidate pyruvate dehydrogenase complex (41) or from Ac~P by the phosphotransacetylase (Pta) (Fig. 1). Unfortunately, efforts to biochemically detect PDHC activity in cell lysates from wild-type and Δ*spxB* D39 strains were unsuccessful using pyruvate as the substrate (≤0.0003 nmol·min^−1^·mg^−1^), in contrast to robust activity (0.015 ± 0.001 nmol·min^−1^·mg^−1^) observed from *E. coli* lysates assayed under the same assay conditions (42). These data thus provide strong genetic evidence that the *spd1025* to *spd1028* genes encode a functional PDHC.

**TABLE 1:** Combination of ΔPDHC (Δ*spd1025* to Δ*spd1028*) and Δ*spxB* mutations is lethal in *S. pneumoniae* D39 strains^a^

Recipient strain	Recipient genotype	Amplicon	No. of colonies
IU1690	D39 (WT)	ΔPDHC::P_c_-(*kan*-*rpsL*^+^)	45
IU2181	D39 *ΔspxB*::P_c_-*erm*	ΔPDHC::P_c_-(*kan*-*rpsL*^+^)	0
		Δ*pbp1b*::P_c_-(*kan*-*rpsL*^+^)	120
IU1781	D39 *rpsL1* (WT)	ΔPDHC::P_c_-(*kan*-*rpsL*^+^)	80
IU2173	D39 *rpsL1 ΔspxB*	ΔPDHC::P_c_-(*kan*-*rpsL*^+^)	0
		Δ*pbp1b*::P_c_-(*kan*-*rpsL*^+^)	~150
IU1781	D39 *rpsL1* (WT)	*ΔspxB*::P_c_-*erm*	>500
IU2793	D39 *rpsL1* ΔPDHC::P_c_-(*kan*-*rpsL*^+^)	*ΔspxB*::P_c_-*erm*	0
		*Δpbp1b*::P_c_-*erm*	~300
IU2950	D39 *rpsL1* ΔPDHC	*ΔspxB*::P_c_-*erm*	0
		*Δpbp1b*::P_c_-*erm*	~300

a Recipient strains were constructed as described in Table S1A, and transformations were performed as described in Materials and Methods. Numbers of colonies obtained from 1-ml transformation mixtures were counted after 24 h of incubation. Amplicons with ~1-kb flanking DNA sequences were obtained from PCRs using templates from the strains and primers listed: ΔPDHC::P_c_-(*kan*-*rpsL*^+^) (IU2793, SR200, and SR201) and *ΔspxB*::P_c_-*erm* (IU2181, SR11, and SR14) and positive-control amplicons Δ*pbp1b*::P_c_-(*kan*-*rpsL*^+^) (K180, P222, and P522) and Δ*pbp1b*::P_c_-*erm* (E193, P222, and P522).

The synthetic lethality of double Δ*spxB* ΔPDHC mutation under aerobic conditions is consistent with the finding that the enzyme responsible for the third acetyl-CoA synthesis pathway, pyruvate formate lyase (PFL), is oxygen sensitive (11) and therefore nonfunctional during aerobic growth. We next investigated the essentiality of PFL during anaerobic growth. A previous study reported two putative *pflB* genes (*spd0235* and *spd0420*), but further informatics and fermentation end product analysis of the single Δ*spd0235* and Δ*spd0420* mutants concluded that *spd0420* encodes PFL (9). The same study also reported that a D39 Δ*pflB* (*spd0420*) mutant lacking pyruvate formate lyase is viable under loosely defined anaerobic conditions established using a rubber-stoppered bottle (9). To investigate the growth of the Δ*pflB* (*spd0420*) strain under strict anaerobic conditions, we streaked out single colonies heavily on TSAII BA plates (Trypticase soy agar II plates containing 5% defibrinated sheep blood) that have been preincubated in an anaerobic hood overnight. We observed that anaerobic growths of single Δ*pflB* and double Δ*pflB* ΔPDHC mutants on TSAII-sheep blood plates were severely inhibited compared to those of D39 wild-type strains. Under aerobic conditions, wild-type D39 parents and Δ*pflB*, ΔPDHC, and double Δ*pflB* ΔPDHC mutants all produced hundreds of colonies on heavily streaked plates, as did the wild type and ΔPDHC mutant under anaerobic conditions. After 24 h of anaerobic incubation, no colonies were observed for the Δ*pflB* and Δ*pflB* ΔPDHC mutants (Table 2). A small number (≤20) of colonies could be obtained with these two strains after 48 h of anaerobic incubation, which suggests that a secondary suppressor mutation or mutations may have arisen. Alternatively, the small numbers of colonies found with the Δ*pflB* (*spd0420*) ΔPDHC mutant under 48-h anaerobic conditions could be the unmasking of a very weak activity of the other putative *pflB* homologue, *spd0235* (9), in the double Δ*pflB* (*spd0420*) ΔPDHC mutants. These results reveal that the PFL pathway encoded by *spd0420* is essential for pneumococcal anaerobic growth (Fig. 1) and is consistent with the report that PDHC is inhibited by NADH under anaerobic conditions (43).

**TABLE 2:** *S. pneumoniae* D39 Δ*pflB* mutants are growth inhibited, and mutants with gene deletions in the *suf* operon are not viable under anaerobic conditions^a^

Strain	Genotype	No. of colonies on plates	
		Anaerobic	Aerobic
IU1690	D39 (WT)	>200	>200 WT size
IU1781	D39 *rpsL1* (WT)	>200	>200 WT size
IU2950	*rpsL1 Δ*PDHC	>200	>200 WT size
IU2822	*ΔpflB*	0	>200 WT size
IU2948	*rpsL1 Δ*PDHC *ΔpflB*	0	>200 WT size
IU3602	*Δspd0091*	>200	>200 WT size
IU3606	*ΔsodA*	>200	>200 WT size
IU3610	*ΔtpxD*	>200	>200 WT size
IU12065	*ΔsufU*	0	>200 small^b^
IU12088	*ΔsufCD*	0	>200 small
IU12090	*ΔsufCDSUB*	0	>200 small

a All strains are of the D39 genetic background and were constructed as described in Table S1A and stored as glycerol stock. Strains were streaked from ice stock onto TSAII BA plates (Fisher Scientific [B21261X]) and incubated at 37°C in an atmosphere of 5% CO_2_ (aerobic condition) for 24 h. For the aerobic condition, a single colony from each strain was heavily streaked onto a TSAII BA plate that had been incubated at 37°C in an atmosphere of 5% CO_2_ for 1 h. For the anaerobic condition, another colony was heavily streaked in the anaerobic hood onto a TSAII BA plate that has been equilibrated in the anaerobic hood for 24 h at room temperature. The numbers of colonies on each plate were examined after 24 h of incubation at 37°C under the 5% CO_2_ condition or under the anaerobic condition at 37°C. The results from this table were obtained from ≥2 independent experiments.

b The colony sizes of the *suf* deletion mutants under the aerobic condition were approximately 1/2 of those of the WT strains.

### Identification of pneumococcal genes potentially involved in the adaptive response to endogenous H_2_O_2_ production.

We next set out to identify other genes beyond *lctO* and *spxB* that are involved in the endogenous H_2_O_2_ production response of *S. pneumoniae*. Microarray analysis was performed comparing relative transcript levels of wild-type *S. pneumoniae* D39 grown aerobically (limited aeration conditions [see Materials and Methods]) versus under strictly anaerobic conditions. Pneumococcus grows well under these aerobic growth conditions, with a doubling time of ≈35 to 40 min, and no lysis is detected until the stationary phase. In contrast, pneumococcus typically grows more slowly and to a far lower growth yield when cultured in a highly aerobic orbital shaking bath at 150 rpm (16). Serially diluted overnight cultures of wild-type strain D39 were grown aerobically to the mid-log phase and diluted into fresh BHI medium preequilibrated with a 5% CO_2_ atmosphere or anaerobically in a Coy anaerobic chamber. Compared to aerobic growths, anaerobically grown cultures typically show a 1-h growth lag but similar doubling times (35 to 45 min) and slightly lower growth yields (optical density at 620 nm [OD_620_] of ~0.6 versus 0.9). The H_2_O_2_ concentration produced by wild-type D39 cultured under these aerobic conditions at mid-log phase (OD_620_ ≈ 0.2) is ≈0.4 mM (data not shown).

We find 40 or 14 genes to be differentially upregulated or downregulated, respectively, when comparing aerobic to anaerobic growth conditions (Table S1D), with a partial list of differentially expressed genes of interest shown in Table 3. The genes that show the highest increase in expression under limited-aeration versus anaerobic conditions are *spd0091*, which encodes a conserved hypothetical protein harboring a rhodanese homology domain (RHD) (44), *tpxD*, encoding a thiol peroxidase (39), *sodA*, encoding a Mn(II) superoxide dismutase, and *spxB*. Additionally, the *spd0762-*to-*spd0766* (*sufC*, *sufD*, *sufS*, *sufU*, and *sufB*) operon, encoding components of a candidate iron-sulfur biogenesis system, and the *piuB-piuD* operon, encoding Fe transporter, show higher expression under limited-aeration conditions. The upregulation of the two iron-related operons may highlight an important role that iron plays in adapting to aerobic growth. Two DNA repair genes, *mutY*, encoding adenine glycosylase active on G-A mispairs, and *ogt*, encoding *O*^6^-methylguanine-DNA methyltransferase (45), show moderate increases in transcription as well. It is also of interest that genes that are involved in acetyl-CoA synthesis pathway are either mildly or moderately upregulated under the limited aeration conditions. In contrast, the operon encoding the anaerobic ribonucleotide reductase NrdDG (*spd0187* to *spd0191*) exhibits lower expression under conditions of limited aeration.

**TABLE 3:** Changes in relative transcript amounts of genes related to oxidative stress in *Streptococcus pneumoniae* D39 grown exponentially in BHI broth with limited aeration compared with growth under the anaerobic condition^a^

Locus tag	Gene; protein description	Fold change^b^
Increased relative expression		
* spd0063^c^*	*strH*; β-*N*-acetyl-hexosaminidase precursor	+3.8
* spd0091*	Codes for conserved hypothetical, rhodanese-like	+16.6
* spd0420*	*pflB*; pyruvate formate-lyase	+2.5
* spd0621*	*lctO*; lactate oxidase	+2.1
* spd0623^d^*	*thiM*; hydroxyethylthiazole kinase	+2.8
* spd0624^d^*	*thiE*; thiamine-phosphate pyrophosphorylase	+2.4
* spd0636^c^*	*spxB*; pyruvate oxidase	+4.2
* spd0667^c^*	*sodA*; Mn(II) superoxide dismutase	+6.5
* spd0762^d^*	*sufC*; Fe-S assembly ATPase	+6.7
* spd0763^d^*	*sufD*; Fe-S assembly factor	+7.3
* spd0764^d^*	*sufS*; cysteine desulfurase	+7.3
* spd0765^d^*	*sufU*; Fe-S assembly factor	+6.6
* spd0766^d^*	*sufB*, Fe-S assembly factor	+4.0
* spd1027^d^*	Pyruvate dehydrogenase E1 component β subunit, candidate	+2.3
* spd1028^d^*	Pyruvate dehydrogenase E1 component α subunit, candidate	+2.6
* spd1086*	*mutY*; similar to A/G-specific adenine glycosylase	+2.4
* spd1287*	*trxB*; thioredoxin reductase	+2.1
* spd1292^d^*	*ogt*; O-6-methylguanine-DNA methyltransferase	+2.5
* spd1464*	*tpxD*; thioredoxin-linked thiol peroxidase	+6.8
* spd1649^c,e^*	*piuB*; Fe ABC transporter, permease	+3.2
* spd1651^c^*	*piuD*; Fe ABC transporter, ATPase	+3.8
Decreased relative expression		
* spd0187*	*nrdD*; anaerobic ribonucleotide reductase	−4.1
* spd0190^d^*	*nrdG*; anaerobic ribonucleotide reductase	−3.9
* spd1041*	*nrdH*; glutaredoxin-like protein	−2.0
* spd1461^d^*	*psaB*; ABC Mn(II) transporter ATP-binding protein	−1.7
* spd1462^d^*	*psaC*; ABC Mn(II) transporter membrane-spanning permease	−1.6
* spd1463^d^*	*psaA*; ABC Mn(II) transporter substrate-binding protein	−2.0

a Growth conditions under limited-aeration and anaerobic conditions and microarray analysis are described in Materials and Methods. Three independent hybridizations for microarray analysis using two independent sets of RNA preparations from *Streptococcus pneumoniae* strain IU1690 (D39) and one RNA set from strain IU1781 (D39 *rpsL1*) were performed. RNA was prepared from exponential cultures grown to an OD_620_ of ≈0.2 under each condition. Fold changes are based on three independent biological replicates, including a dye swap. This table lists genes related to oxidative stress and which show more than 2-fold changes in expression level with Bayesian *P* values of <0.001. *psaB* and *psaC* are listed to show their coregulation with *psaA*, although the fold changes are less than 2. A complete set of genes, including 40 upregulated and 14 downregulated genes with >2-fold changes and *P* values of <0.001 are listed in Table S1D. Intensity and expression ratio data for all transcripts have been deposited in the GEO database (GenBank accession no. GSE19791).

b + or −, upregulated (+) or downregulated (−) under the limited-aeration condition.

c Members of the *spxR* regulon (15).

d Genes in an operon coregulated with the adjacent gene.

e Members of the *ritR* regulon (24).

A previous study (23) using an unencapsulated D39 derivative of the laboratory R6 strain and highly aerobic and less anaerobic (GasPak-induced) growth conditions than our present study revealed differential expression of 69 genes in aerobiosis compared with anaerobiosis. The genes that are common between the two studies are the upregulation of *spd0091*, *sodA*, *tpxD*, *thiM* (thiamine-phosphate pyrophosphorylase), and *spd1588* (hypothetical protein) and the downregulation of the operon *spd0187* to *spd0191*, encoding NrdD and NrdG, under aerobiosis compared to anaerobiosis conditions. The genes that showed opposite trends in the two studies are *pflB*, *piuB*, and *piuD*, which showed increased expression in our study but decreased expression in Bortoni’s study under aerobic conditions (23). Interestingly, *rgg*, encoding a putative oxidative stress-sensing transcriptional regulator, was shown to be highly expressed under the aerobic conditions in Bortoni’s study but was unchanged in our study. In contrast, four members of the *spxR* regulon (15), *spxB*, *strH*, *piuB*, and *piuD*, were upregulated in our study but either did not change in expression (*spxB* and *strH*) or were downregulated (*piuB* and *piuD*) in Bortoni’s study. It is interesting to note that *strH* encodes an important exoglycosidase implicated in colonization in the airway (46).

Notably, many genes or regulons that were implicated in coping with exogenous oxidative stress were not differentially expressed in our study of the endogenous oxidative stress response. They include *ritR* (iron regulator RitR [SPD_0344]) (24), *ritR*-regulated *dpr* (nonheme iron-containing ferritin) (47), *nmlR* (SPD_1637) (25, 48), *nmlR*-regulated *adhC* (formaldehyde dehydrogenase) (25), *ciaRH* (27), *ciaRH* regulon member *htrA* (27), *clpP* (49), *rgg* (putative oxidative transcriptional regulator) (23), *etrx1* (*spd0571* and *spd0572*), and *etrx2* (*spd0885* and *spd0886*), operons encoding cell surface thioredoxin-fold lipoproteins implicated in repair of methionine sulfoxide adducts (50). This suggests that the ambient responses to endogenous H_2_O_2_ production are distinct from those resulting from acute exogenous oxidative stress.

### Protein sulfenylation in *S. pneumoniae* is limited to a small number of major protein targets and is correlated with the cellular H_2_O_2_ load.

**FIG 3:**
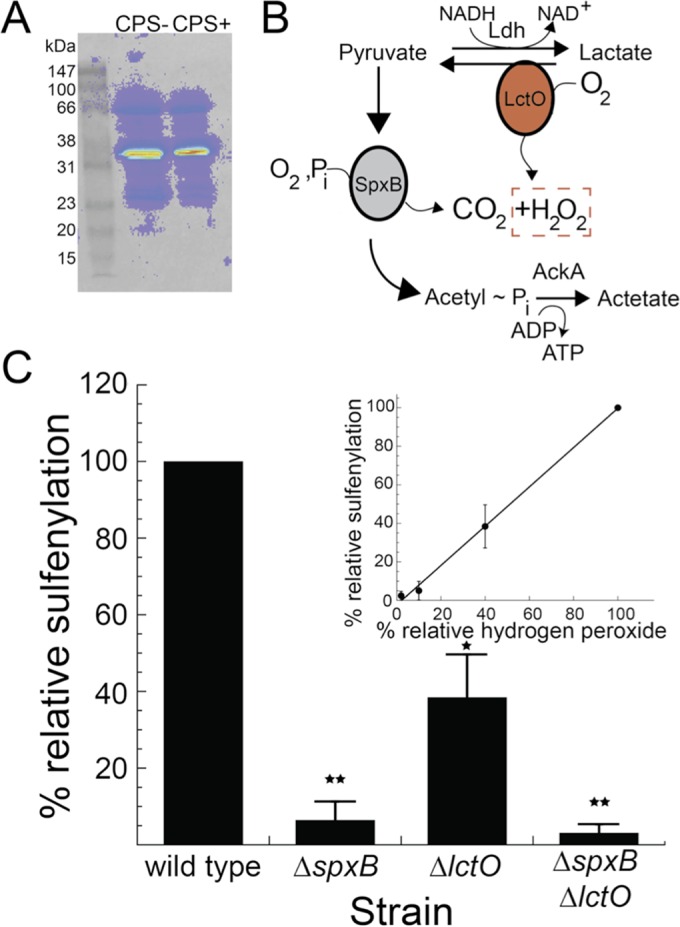
Proteome sulfenylation levels correlate with endogenous hydrogen peroxide (H_2_O_2_) levels in cells. (A) Representative sulfenylation profile of encapsulated (CPS*+* [IU1781]) versus unencapulsated (CPS− [IU1945]) *Streptococcus pneumoniae* D39 (*S. pneumoniae*) strains grown in a rich medium (BHI) after labeling with 10 mM dimedone for 1 h in cell culture. Labeling was visualized using antibodies to 2-thiodimedone on soluble proteins via Western blotting. (B) Schematic illustration of hydrogen peroxide-generating enzymes in *S. pneumoniae*. SpxB, pyruvate oxidase; LctO, lactate oxidase; AckA, acetate kinase. (C) *S. pneumoniae* GapA sulfenylation is modulated by SpxB and LctO activity and correlates with the relative H_2_O_2_ concentrations (inset). *, *P* < 0.05, and **, *P* < 0.005, based on one-sample *t* test.

The major reversible posttranslational modification of the proteome that is expected to occur in the presence of H_2_O_2_ is sulfenylation (*S*-hydroxylation) of solvent-exposed protein thiols (32, 51). Compared to *Escherichia coli*, *S. pneumoniae* produces 19,000-fold more endogenous H_2_O_2_ (≈20 nM versus 380 ± 40 µM H_2_O_2_) (52) (data not shown). This highlights the significant endogenous stress that the pneumococcus endures during aerobic growth. Proteome sulfenylation profiles of whole lysates from wild-type *S. pneumoniae* D39 (*cps*^+^) and cells lacking the polysaccharide capsule (*Δcps*) incubated with 5,5-dimethyl-1,3-cyclohexanedione (dimedone) to capture sulfenylated cysteines on proteins (28, 53) reveals a relatively limited number of major H_2_O_2_ targets (Fig. 3A), independent of capsule production. The major band at ≈36 kDa corresponds to the glycolytic enzyme glyceraldehyde-3-phosphate dehydrogenase (GapA) (Fig. 1), with the other major sulfenylation target at ≈66 kDa, SpxB (Fig. 3A). Strikingly, we find that proteome sulfenylation reflects the total H_2_O_2_ burden imposed by each H_2_O_2_-producing strain (Fig. 3B and C), revealing that sulfenylation of GapA can be used as a readout for total intracellular H_2_O_2_ loads.

### TpxD controls the level of endogenous protein sulfenylation.

**FIG 4:**
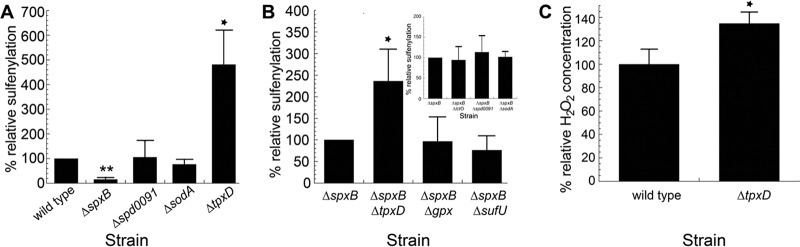
TpxD controls proteome sulfenylation levels in *S. pneumoniae*. (A) Relative *S. pneumoniae* GapA sulfenylation profiles of various deletion strains of *S. pneumoniae* relative to the wild-type (IU1690) strain. The deletion strains have the genotypes Δ*spxB* (IU2181), Δ*spd0091* (IU3602), Δ*sodA* (IU3606), and Δ*tpxD* (IU3610) (Table S1A). (B) Relative *S. pneumoniae* GapA sulfenylation levels in various double deletion strains of *S. pneumoniae* relative to the Δ*spxB* (IU2173) strain. The strains tested have the genotypes Δ*spxB ΔtpxD* (IU3611), Δ*spxB* Δ*gpx* (IU3614), Δ*spxB ΔsufU* (IU3617), Δ*spxB ΔlctO* (IU3284), Δ*spxB Δspd0091* (IU3603), and Δ*spxB ΔsodA* (IU3607). (C) Relative H_2_O_2_ concentration in wild type (IU1690) versus Δ*tpxD* (IU3610) *S. pneumoniae* strains measured under our microaerophilic conditions. *, *P* ≤ 0.05, and **, *P* ≤ 0.005, based on a one-sample *t* test.

Inspection of the microarray data (Table 3 and Table S1D) suggests that several of the genes induced by aerobic growth, and therefore increased by endogenous H_2_O_2_ stress, may contribute to decreasing endogenous proteome sulfenylation. Proteome sulfenylation profiles obtained with *sodA*, *spd0091*, *tpxD*, *gpx*, and *sufU* deletion strains reveal that only the *ΔtpxD* strain results in a change in proteome sulfenylation, with an ~5-fold increase compared to the isogenic wild-type strain or isogenic Δ*spxB* strain (Fig. 4A and B; see Fig. S1 in the supplemental material). Sulfenylation levels are also higher when *tpxD* is deleted in a Δ*spxB* background (Fig. 4B; Fig. S1). These increases in cellular sulfenylation in the Δ*tpxD* strains are not solely attributed to the correspondingly increase in H_2_O_2_ production, since the Δ*tpxD* strain exhibits just a 30% increase in measurable H_2_O_2_ relative to the wild-type strain (Fig. 4C) (39). These findings reveal that Δ*tpxD* strains are impaired in global control of proteome sulfenylation under aerobic conditions. Increased proteome sulfenylation may negatively impact fitness during infection, since pneumococcal cells lacking *tpxD* are less virulent in mouse models of infection (39).

10.1128/mSphere.00291-16.2 FIG S1  Representative proteome sulfenylation profiles of *S. pneumoniae* D39 strains after labeling with dimedone. Profiling was visualized utilizing various mutant strains expected to vary the amount of endogenously produced H_2_O_2_ (upper left panel) showing reduction of intensity correlating well with the relative hydrogen peroxide concentration. The remaining panels show various mutants under these conditions to identify proteins that protect *S. pneumoniae* from endogenous hydrogen peroxide. These data are presented in graphical form in Fig. 4. The band at ≈66 kDa is identified as SpxB since it is absent in Δ*spxB* strains (65.2 kDa), and the band at ≈36 kDa is identified as GapA (35.8 kDa) (see Table S2C). Download Figure S1, PDF file, 0.1 MB. Copyright © 2017 Lisher et al. 2017 Lisher et al. This content is distributed under the terms of the Creative Commons Attribution 4.0 International license.

### Extracellular metal stresses impact protein sulfenylation in distinct ways.

Metal homeostasis systems are integrally connected to the oxidative stress response in bacteria (54), and *tpxD*, encoded downstream of *psaBCA*, is reported to modulate transcription of the Mn import genes in the pneumococcus (39). We therefore obtained whole-lysate sulfenylation profiles in the presence of exogenous transition metal (Cu, Zn, Mn, or Fe) using concentrations sufficient (≥200 to 500 µM) to repress transcription of uptake genes and induce the expression of efflux transporters (55–58) (see Fig. S2 in the supplemental material). The more thiophilic metals Cu and Zn (to a lesser extent) protect proteome thiols from sulfenylation (Fig. S2A and E). Fe(III) addition leads to a small increase in the spectrum of sulfenylated proteins (Fig. S2C) but has no significant impact on the sulfenylation status of GapA (Fig. S2A).

10.1128/mSphere.00291-16.3 FIG S2  Effect of transition metal stress on sulfenylation levels of wild-type and mutant *S. pneumoniae* D39 strains. The relative intensity of the ≈36-kDa band (GapA) is quantified for each stress condition from triplicate biological replicates. (A) Effect of Zn (0.2 mM), Cu (0.5 mM), and Fe(III) (0.05 mM) stresses on cellular sulfenylation and (B) effect of no or 0.1 mM Mn(II) added to cultures of the wild-type (IU1781), Δ*mntE* (IU4024), and Δ*psaR* (IU6745) strains (57) in cells grown without an Fe chelator (gray bars) and with the Fe chelator DFO (red bars). *, *P* < 0.05 based on one-sample *t* test. Corresponding Western blots for this graphical summary are shown in panels C to E as representative sulfenylation profiles of various D39 strains of *S. pneumoniae* under metal stress. (C) Sulfenylation profiles of wild-type, Δ*psaR*, and Δ*mntE* strains ± 0.1 mM Mn(II). (D) Sulfenylation profiles of wild-type, Δ*psaR*, and Δ*mntE* strains + 15 µM DFOM ± 0.1 mM Mn(II). (E) Sulfenylation profiles of wild-type strains under extracellular metal stresses: BHI (lane 1), 0.2 mM Zn(II) (lane 2), 0.5 mM Cu(II) (lane 3), or 0.05 mM Fe(III) (lane 4). The strains utilized are IU1781, IU6745, and IU4024 for the wild type and Δ*psaR* and Δ*mntE* mutants, respectively. Download Figure S2, PDF file, 0.4 MB. Copyright © 2017 Lisher et al. 2017 Lisher et al. This content is distributed under the terms of the Creative Commons Attribution 4.0 International license.

Mn, on the other hand, is reported to protect *S. pneumoniae* from external ROS (59) and therefore might be anticipated to reduce cellular sulfenylation levels. Mn homeostasis in *S. pneumoniae* is governed by the activities of the Mn-specific importer PsaBCA and the Mn exporter MntE; *psaBC*A transcription is repressed by Mn-bound PsaR, thereby limiting Mn import under Mn-replete conditions (60). Mn-stressed cells have increased Fe levels (J. Martin, submitted for publication). Cellular sulfenylation levels during Mn overload increase ≈40% for all strains harboring Mn homeostasis mutants (Fig. S2B). This Mn-overload-dependent increase in sulfenylation is abrogated by addition of the Fe-specific chelator desferrioxamine (DFO) (Fig. S2B), suggesting that Mn overload leads to increased bioavailable Fe that is responsible for an increase in H_2_O_2_-mediated sulfenylation. Examination of the total cell-associated Mn and Fe contents of cultures grown with DFO supports this hypothesis and suggests that a Mn-dependent increase in Fe levels impacts proteome sulfenylation levels (see Fig. S3 in the supplemental material). How increased Fe leads to increased sulfenylation is not yet known given that soluble Fe would tend to consume H_2_O_2_, leading to increased hydroxyl radical via Fenton chemistry, and potentially proteome thiyl radical formation.

10.1128/mSphere.00291-16.4 FIG S3  Iron (Fe) chelation limits the Mn-dependent increase in total cell-associated Fe content. Culture aliquots were sampled at an OD_620_ of ~0.2, and the total cell-associated metal content was measured for unstressed (black, light gray, and dark gray bars) and stressed (dark red, red, and orange bars) strains (0.1 mM Mn). The total cell-associated metal content was measured for the wild-type (black and dark red), Δ*psaR* (light gray and red), and Δ*mntE* (dark gray and orange) strains. The strains utilized are IU1781, IU6745, and IU4024 for the wild type and Δ*psaR* and Δ*mntE* mutants, respectively. Error bars show the standard error of the mean (±SEM) from three independent growths. Download Figure S3, PDF file, 0.1 MB. Copyright © 2017 Lisher et al. 2017 Lisher et al. This content is distributed under the terms of the Creative Commons Attribution 4.0 International license.

### SpxB, glycolytic, capsule, and nucleotide biosynthesis enzymes are targets of protein sulfenylation by endogenous H_2_O_2_.

**FIG 5:**
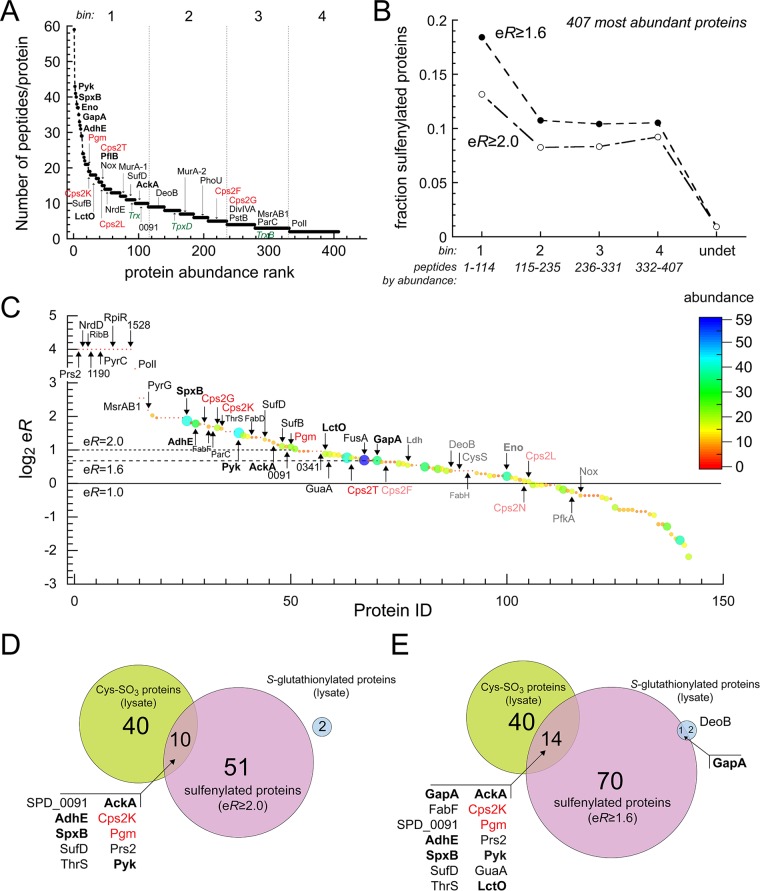
Proteomic profiling of sulfenylation by endogenous H_2_O_2_ in *S. pneumoniae* cells. (A) The 407 total peptides confidently identified (≥2 unique peptides per protein) in an unenriched total cell lysate of *S. pneumoniae* D39 are ranked according to unique peptide abundance (62) and binned into the four bins numbered at the top (also see panel B). A subset of proteins are highlighted by protein identification. Boldface black indicates enzymes of glycolysis and the pyruvate node (Fig. 1), red indicates enzymes of capsule biosynthesis (Fig. 6), and green indicates thioredoxin (Trx), thioredoxin reductase (TrxB), and thiol peroxidase (TpxD), not detected as sulfenylated and shown for reference. (B) The fraction of sulfenylated proteins as a function of protein abundance for enrichment levels (eR) of 1.6 (dashed line; 75.6% confidence [Fig. S5]) or 2.0 (dot-dash line; 80.6% confidence [Fig. S5]). Bins: 1, 10 to 59 unique peptides per protein (114 total proteins); 2, 5 to 9 unique peptides per protein (121 total proteins); 3, 3 to 4 unique peptides per protein (96 total proteins); 4, 2 unique peptides per protein (76 total proteins). undet, undetected in cells. See Table S2A and B for a complete list of all 407 most abundant proteins. (C) Plot of enrichment ratio (eR) versus arbitrary Protein ID index, ranked from largest to smallest values of log_2_ eR. Proteins detected only in the DAz-2-enriched eluant and not in the total lysate were arbitrarily assigned a log_2_ eR value of 4.0 (13 proteins). Lines corresponding to eR values of 1.0 (no enrichment), 1.6 (all proteins enriched relative to GapA, a known sulfenylation target), and 2.0 are shown for reference. Selected proteins are indicated. Boldface black indicates enzymes of glycolysis and the pyruvate node (Fig. 1), and red indicates enzymes of capsule biosynthesis (Fig. 6). Each protein ID symbol is colored and sized according to cellular abundance (number of unique peptides recovered as proxy for abundance). See Table S2A and B for a complete list of all 142 Cys-containing proteins detected. Venn diagrams compare the number of sulfenylated proteins in panels D (eR ≥ 2.0) and E (eR ≥ 1.6) versus the number of Cys-sulfonylated proteins and number of *S*-glutathionylated proteins in an unenriched total lysate. All 10 (D) and 14 (E) sulfonylated proteins are identified with high confidence as sulfenylated in cells (eR ≥ 1.6), as is one of the two *S*-glutathionylated targets (GapA) (Table S2D and E). Names of proteins are colored as in panel C.

In order to further evaluate the effect of endogenous sulfenylation on *S. pneumoniae* metabolism, we sought to identify additional targets of protein sulfenylation utilizing a streptavidin enrichment-based approach (37). Here, whole-cell lysates were obtained from cells grown with an azide-derivatized dimedone, DAz-2, and proteins conjugated to an alkyne-biotin utilizing Cu(I)-catalyzed 1,3-dipolar cycloaddition (61). Biotinylated proteins were enriched on NeutrAvidin beads and analyzed by mass spectrometry. Sulfenylated proteins were identified by elution of biotinylated proteins from the extensively washed beads by boiling in 1× Laemmli buffer and running the samples on an SDS-PAGE gel followed by silver staining (see Fig. S4A in the supplemental material). Regions of the gel were then excised and subjected to in-gel tryptic digestion and identified by liquid chromatography-tandem mass spectrometry (LC-MS/MS) (see Table S2A and B in the supplemental material). Analysis of a parallel control sample of unlabeled lysate worked up in the same way reveals very limited overlap to proteins in the DAz-2-enriched sample (Fig. S4B; Table S2A and B). We compared the list of proteins recovered from these two samples to an unfractionated analysis of the total cell lysate, also subjected to tryptic digestion and analyzed by bottom-up LC-MS/MS (Fig. 5A; Table S2A and B). We then calculated an enrichment ratio (eR), defined by the ratio of the fractional abundance of a particular protein in the DAz-2-eluted sample to the fractional abundance in the unfractionated cell lysate. Fractional abundance in each faction was determined using a label-free approach that designates the number of unique tryptic peptides obtained for each protein as a proxy for cellular abundance (62); furthermore, we considered an identification positive only if two or more peptides could be matched to a particular protein (Table S2C).

10.1128/mSphere.00291-16.5 FIG S4  Gels for the identification of sulfenylated proteins from (A) DAz-2-labeled whole lysates, (B) control whole lysates of *S. pneumoniae* through the biotin enrichment and elution steps as outlined in panel A, and (C) purified *S. pneumoniae* GapA and *S. pneumoniae* SpxB. Boxed areas show the excised gel sections (P1 to P6) that were analyzed by in-gel tryptic digestion and LC-MS/MS. The major bands in the P2 and P4 fractions in panel A, lane E (marked with red asterisks), are SpxB (65.2 kDa) and GapA (35.9 kDa), respectively. Load, column load; FT, column flowthrough; W1 to W4, wash fractions 1 to 4; E, eluate (see Materials and Methods). A summary of these peptide data are provided in Table S2A and B and analyzed as indicated in Table S2C (Fig. 5). Download Figure S4, PDF file, 0.1 MB. Copyright © 2017 Lisher et al. 2017 Lisher et al. This content is distributed under the terms of the Creative Commons Attribution 4.0 International license.

10.1128/mSphere.00291-16.6 TABLE S2  Proteomics profiling of thiol redox proteome of *S. pneumoniae* D39. (A) Listing of all tryptic peptides recovered from silver-stained SDS-PAGE gel slices derived from Daz-2 and control-eluted NeutrAvidin fractions compared to a total unfractionated cell lysate. (B) Listing of any tryptic peptide recovered from silver-stained SDS-PAGE gel slices derived from Daz-2, control-eluted NeutrAvidin fractions, or total unfractionated cell lysate. (C) Summary of the recovery of peptides and proteins from an unfractionated total lysate and DAz-2 and control elutions from the NeutrAvidin beads. (D) List of oxidative PTMs on proteins detected in an unfractionated lysate and detected as sulfenylated in cells (eR ≥ 1.6 [Fig. 5C]). (E) Listing of all peptides identified in the whole-cell lysate with oxidized or glutathionylated cysteine residues. (F) Summary of candidate Fe-S proteins in *S. pneumoniae* D39 identified by a bioinformatics search using IronSulfurProteoHome (77). Download Table S2, XLS file, 0.2 MB. Copyright © 2017 Lisher et al. 2017 Lisher et al. This content is distributed under the terms of the Creative Commons Attribution 4.0 International license.

A total of 407 of the 1,914 predicted proteins (21.2%) can be detected in an unfractionated cell lysate, with 142 Cys-containing proteins and 54 non-Cys-containing proteins found in the DAz-2-eluted fraction. eR factors were found to vary from infinite (13 proteins found only in the DAz-2-enriched fraction [Fig. 5B and C]) to ≈0.2, with eR values of ≥1.6 (just below that of the known sulfenylation target GapA [eR = 1.61]) and ≥2.0 (Fig. 5C), identified as sulfenylated with 75% and 80% confidence, respectively (see Fig. S5 in the supplemental material). These eR values give 70 and 51 unique proteins, respectively, denoted as sulfenylated in cells. Major sulfenylation targets are the glycolytic enzymes GapA and pyruvate kinase (Pyk) (Fig. 1) and thus serve as positive controls in this experiment, since these enzymes have previously been identified as harboring oxidation-sensitive cysteines in eukaryotic cells (63, 64). Approximately 10 to 15% of all proteins detected in the unfractionated cell lysate are identified as sulfenylated in cells (Fig. 5B), revealing that this modification is widespread in the proteome.

10.1128/mSphere.00291-16.7 FIG S5  Enrichment ratios (eR) calculated for the 54 non-cysteine-containing proteins obtained in the SDS eluate of the NeutrAvidin-DAz-2-biotin column (Fig. S4A, lane E). (A) Plot of log_2_ eR as a function of the Protein ID index, ranked from largest to smallest eR. A log_2_ eR of 4.0 corresponds to the three proteins that were found only in the DAz-2 eluate and not in the total cell lysate. All other eR represent % of abundance in DAz-2 eluate/% of abundance in a cell lysate, where percentage is defined by (no. of peptides obtained for each protein/total no. of peptides detected in that sample) × 100 (see Table S2A and B). Proteins highlighted in italic were identified in the control lysate (Fig. S2B, line E). A total of 20/54 peptides are derived from the cellular translation machinery (see Table S2A and B for a complete list) and are indicative of nonspecific binding to the resin. This plot is to be compared to Fig. 5C for Cys-containing DAz-2-eluted proteins. (B) Histogram plot of the 142 Cys-containing peptides identified in the DAz-2 eluate (gray) versus 54 non-cysteine-containing peptides (red) binned in log_2_ eR = 0.4 bin and fit to a Gaussian function with identical limits (log_2_ eRs ranging from −3 to 3). Gray bars go to zero and reflect total counts, as do red bars positioned in front of gray bars. The peptides in log_2_ eR = 4.0 correspond to those 13 (46 peptides total [Table S2A and B]) and 3 (7 peptides total [Table S2A and B]) Cys- and non-Cys-containing proteins, respectively, that are only found in the DAz-2 eluant and were not contained in the fit. The maximum values of log_2_ eR are 0.54 ± 0.07 and −0.12 ± 0.09 for the Cys- and non-Cys-containing proteins, respectively. Note that a Gaussian function centered around 0 is as expected for the nonspecific binding of non-Cys-containing peptides to the resin. eRs of 2.0 and 1.6 for Cys-containing peptides give false-positive values of 19.4% and 24.4%, respectively, indicative of ≈80% and ≈75% confidence that the 51 and 70 proteins in the DAz-2 eluate (see Table S2A and B for a complete list of proteins and Fig. 5D and E) are sulfenylated in cells and captured by the dimedone reagent. Download Figure S5, PDF file, 0.1 MB. Copyright © 2017 Lisher et al. 2017 Lisher et al. This content is distributed under the terms of the Creative Commons Attribution 4.0 International license.

**FIG 6:**
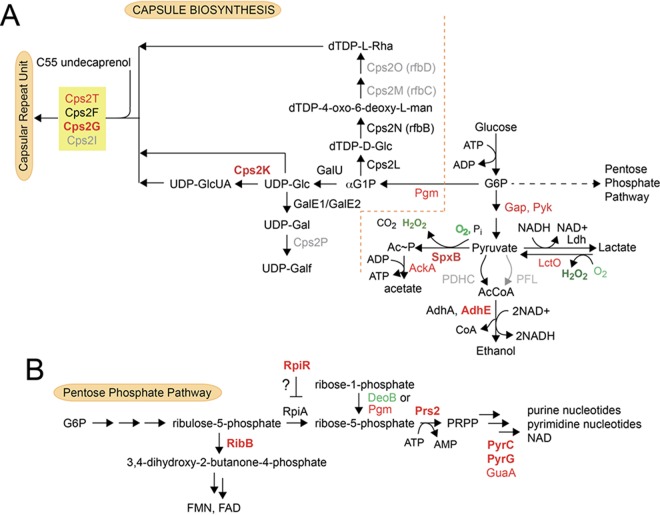
Schematic illustration of polysaccharide capsule biosynthesis (A) and nucleotide/cofactor biosynthesis (B) in* S. pneumoniae* D39 and its relationship to glycolysis and the fate of pyruvate under aerobic conditions. The enzymes that are found to be sulfenylated (eR ≥ 1.6, GapA) are highlighted in red, with bold red corresponding to those proteins with eR ≥ 3.0. Black indicates protein was detected in the total cell lysate, but not enriched, and gray indicates the protein was not detected in the cell lysate. Two of the three glycosyltransferases known to be involved in the synthesis of the capsule repeat unit (Cps2T and Cps2G; Cps2F is just below eR for GapA [Fig. 5C]) (65) and CpsK are all identified as sulfenylated in cells with high confidence. In panel B, key enzymes associated with synthesis and utilization of ribose-5-phosphate and sulfenylated in cells are indicated, as highlighted in panel A. Green indicates *S*-glutathionylated in the cell lysate (DeoB).

Strikingly, nearly all enzymes of the pyruvate node (Fig. 6A), including LctO, SpxB, acetate kinase (AckA), and the major Fe-dependent bifunctional alcohol dehydrogenase (AdhE), are sulfenylated in cells (eR ≥ 1.6). In addition to these enzymes, a number of proteins involved in cell division and replication (PolI, ParC, and DivIVA), Fe-S cluster biogenesis, or sulfur metabolism (SufB, SufD, and SPD_0091; all transcriptionally upregulated upon a shift from anaerobic to aerobic conditions [Table 3]), and the redox stress response (MsrAB1 [methionine sulfoxide reductase]) are sulfenylated in cells. In addition, 13 low-abundance proteins not detected in the cell lysate and found only in the DAz-2-enriched fraction (designated log_2_ eR = 4 [Fig. 5C]) were identified. These proteins are potential regulatory candidates and include SPD_1190, an uncharacterized aminohydrolase superfamily member with some similarity to adenosine deaminase, and PyrC (dihydrooratase [eR = 1]), which along with PyrG (CTP synthase [eR = 4.5]), are involved in *de novo* pyrimidine biosynthesis. Prs2 (eR = 1), the ribose-5-phosphate pyrophosphokinase, and GuaA (GMP synthetase [eR = 1.8]) are also involved in nucleotide and purine biosynthesis, respectively; in addition, an uncharacterized candidate ribose-responsive and pentose phosphate pathway (PPP) transcriptional regulator, RpiR, gives an eR of 1. These data suggest that sulfenylation may also impact nucleotide biosynthesis and its regulation (Fig. 6B). Finally, the anaerobic ribonucleotide reductase NrdD, whose transcription is repressed under aerobic conditions (Table 2), may be further inactivated by sulfenylation (eR = 1).

In addition to the major enzymes of glycolysis and pyruvate metabolism, no fewer than four enzymes, including the phosphoglucomutase (Pgm) that isomerizes glucose-6-phosphate to glucose-1-phosphate, the immediate precursor to the three major classes of nucleotide diphosphate-activated monosaccharide precursors, UDP-Glc, UDP-glucuronate (GlcUA), and dTDP-l-rhamnose (Rha), are identified as cellular sulfenylation targets (Fig. 5C). Cps2K, which converts UDP-Glc to UDP-GlcUA, is particularly interesting since the only Cys residue in the molecule is the catalytic Cys259 and is among the most highly sulfenylated proteins in cells (Fig. 5C). These activated sugars are substrates for the four glucosyltransferases (Cps2T, Cps2F, Cps2G, and Cps2I) that add the capsular repeat oligosaccharide structure onto the C55-undecaprenol pyrophosphoryl-Glc (Fig. 6A). Strikingly, three glycosyltransferases, including the low-abundance Cps2G (Fig. 5A) and Cps2T, reported to catalyze the rate-determining and committed steps in capsular repeat synthesis (65, 66), respectively, are sulfenylated in cells.

Although sulfenylation (Cys-SOH) is the major reversible thiol oxidative modification in cells, *S*-glutathionylation of sulfenylated Cys and irreversible hyperoxidation to sulfinylated (Cys-SO_2_) and sulfonylated (Cys-SO_3_) cysteines are also possible under these conditions. We therefore queried our unfractionated lysate for evidence of these modifications (Table S2D and E; Fig. 5D and E). We find that 40 proteins in the lysate (≈10% of the lysate; 41% of Cys-containing proteins) are sulfonylated, and these include 10 (eR ≥ 2.0) or 14 (eR ≥ 1.6) sulfenylated proteins, including major targets of the pyruvate metabolic node and capsule biosynthesis and those genes upregulated under aerobic conditions (SPD_0091 and SufD) (Fig. 5D and E). In addition, the resolving Cys of glutathione reductase (Gor) is sulfonylated, while GapA and DeoB, a phosphopentose mutase structurally homologous to Pgm, and responsible for converting ribose-1-P to ribose-5-P (the substrate for sulfenylation target Prs2 [Fig. 6B]), are *S*-glutathionylated in cells. We show below that GapA *S*-glutathionylation is inhibitory; interestingly, DeoB *S*-glutathionylation may also be regulatory since the modified Cys is very close to the active site (67) (Table S2D).

### Effect of sulfenylation of *S. pneumoniae* GapA on activity.

**FIG 7:**
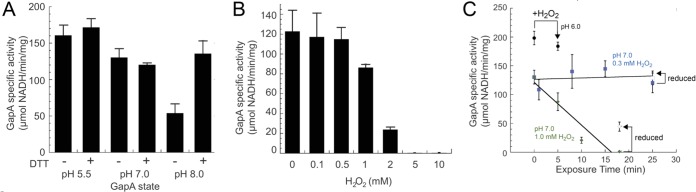
*S. pneumoniae* GapA is sensitive to oxygen and H_2_O_2_ exposure. (A) Specific activity of recombinant *S. pneumoniae* GapA at various pHs. (B) Specific activity of recombinant *S. pneumoniae* GapA after incubation with H_2_O_2_ (0 to 10 mM) at pH 7.0. (C) Specific activity of *S. pneumoniae* GapA highlighting the role of pH and H_2_O_2_ concentration. *S. pneumoniae* GapA is resistant to H_2_O_2_ at pH 6.0 (black circles). In the blue squares, the time-dependent inactivation of recombinant *S. pneumoniae* GapA is shown in the presence 0.3 mM H_2_O_2_ at pH 7.0. Green diamonds show time-dependent inactivation of recombinant *S. pneumoniae* GapA at pH 7.0 in the presence of 1 mM H_2_O_2_. Open symbols show the specific activity of *S. pneumoniae* GapA after incubation with DTT (50 mM).

To further investigate the functional impact of sulfenylation of major targets in *S. pneumoniae*, we purified pneumococcal GapA and SpxB and characterized their enzymatic activities. As expected for an enzyme with a catalytic thiol, *S. pneumoniae* GapA activity is highly pH dependent, and the absence of reducing agent at pH 8.0 leads to a significant, reversible decrease in activity (Fig. 7A and B). At pH 6.0, *S. pneumoniae* GapA is completely resistant to H_2_O_2_ (Fig. 7C); at pH 7.0, however, a short pulse (5 min) of 2 mM H_2_O_2_ reduces GapA activity to 20% of the initial activity, with higher concentrations of H_2_O_2_ completely inhibitory (Fig. 7B). However, exposure of GapA to a physiologically relevant H_2_O_2_ concentration of 0.3 mM at pH 7.0 reveals that the enzyme is relatively resistant to inactivation up to 25 min but is completely inactivated by 1 mM H_2_O_2_ after 18 min (Fig. 7C), much of it irreversibly, as a result of formation of higher oxidation states (Table S2D).

We next addressed if purified TpxD thiol peroxidase, in conjunction with the *S. pneumoniae* thioredoxin (TrxA)-thioredoxin reductase (TrxB) system (68), is capable of directly repairing sulfenylated GapA as a model substrate or simply functions to reduce H_2_O_2_ to H_2_O. Although the addition of 10 mM H_2_O_2_ to purified *S. pneumoniae* TpxD, TrxA, and TrxB gives rise to robust hydroperoxidase activity, as previously reported (see Fig. S6A in the supplemental material) (39), we find no recovery of enzyme activity when the TpxD-TrxA-TrxB system is incubated with freshly sulfenylated and desalted *S. pneumoniae* GapA (Fig. S6B). We also show that sulfenylated GapA is readily *S*-glutathionylated at the catalytic Cys upon incubation with reduced glutathione (Fig. S6D), and as expected, formation of this mixed disulfide abolishes activity (Fig. S6C). This adduct is detected in lysates (Tables S2D and E) and is likely repaired in cells by an as-yet-unidentified glutaredoxin system (see Fig. S7A) (30) and thus ultimately prevents irreversible oxidative modifications of protein thiols (69). We conclude that abundant LMW thiols are critical for resolving H_2_O_2_-induced protein sulfenylation in *S. pneumoniae*, with the primary role of the TpxD-TrxA-TrxB system to enable clearance of H_2_O_2_ directly so as to limit the extent of proteome sulfenylation and likely other thiol oxidative modifications detected here.

10.1128/mSphere.00291-16.8 FIG S6  Low-molecular-weight thiols are capable of repairing sulfenylated *S. pneumoniae* GapA *in vitro*. (A) NADPH-dependent peroxidase activity of recombinant *S. pneumoniae* TpxD (2.5 µM) in the presence of 1 mM H_2_O_2_, 100 µM TrxA, 50 µM TrxB, and 0.2 mM NADPH. (B) Specific activity of sulfenylated *S. pneumoniae* GapA in the presence of DTT and the TpxD peroxidase-thioredoxin-thioredoxin reductase repair system (2.5 µM TpxD, 100 µM TrxA, 50 µM TrxB, 200 µM NADPH) following oxidation by H_2_O_2_ (2 mM, 5 min). (C) Specific activity of sulfenylated *S. pneumoniae* GapA following incubation with excess DTT (50 mM), l-cysteine (50 mM), and reduced glutathione (50 mM). *, *P* < 0.05 based on 2-tailed unpaired *t* test. (D) LC-MS/MS analysis of the GapA tryptic peptide (residues 129 to 161; 33 residues, +3 charge state) harboring an *S*-glutathionylation modification at the active site Cys, C151, with C155 capped by iodoacetamide (CAM). Note that the same modification was detected in an unenriched cell lysate (Table S2A and B). Download Figure S6, PDF file, 0.1 MB. Copyright © 2017 Lisher et al. 2017 Lisher et al. This content is distributed under the terms of the Creative Commons Attribution 4.0 International license.

10.1128/mSphere.00291-16.9 FIG S7  (A) Schematic of the putative endogenous H_2_O_2_ stress system in *Streptococcus pneumoniae*. H_2_O_2_ is detoxified through the activities of the thiol peroxidase TpxD and the putative glutathione peroxidase Gpx, leading to water and an oxidized glutathione disulfide in the case of Gpx. Glutathione can be utilized to reduce sulfenylated proteins through *S*-glutathionylation and reduction by a putative as yet unknown glutaredoxin, reduced glutathione, and the glutathione reductase (Gor) (B). Ribbon representation of the structure of Lpg2838 from *L. pneumophilia* (PDB 4F67, 254 residues; 12 to 244 in the model) is related (39% identical, 58% similarity) to the N-terminal 244 residues of SPD_0091. The molecule is characterized by an N-terminal α-β sandwich domain (yellow), followed by a linker (cyan) and a canonical rhodanese homology domain (RHD) (salmon), followed by an unstructured C-terminal domain (blue). SPD_0091 harbors an additional C-terminal domain (residues 230 to 322) annotated as a rhodanese-like domain that lacks an active site Cys, not found in Lpg2838. The RHD contains a conserved C177TGGIRC182 sequence, with the first Cys177 typical of a rhodanese (82) and the second C182 disulfide bonded to the C177. Both Cys residues are shown in space-fill. It is unclear from this structure the degree to which the two globular domains interact in solution, in which this linker considerably shorter and not conserved in SPD_0091. Download Figure S7, PDF file, 0.8 MB. Copyright © 2017 Lisher et al. 2017 Lisher et al. This content is distributed under the terms of the Creative Commons Attribution 4.0 International license.

### *S. pneumoniae* SpxB activity is not modulated by H_2_O_2_-mediated sulfenylation.

**FIG 8:**
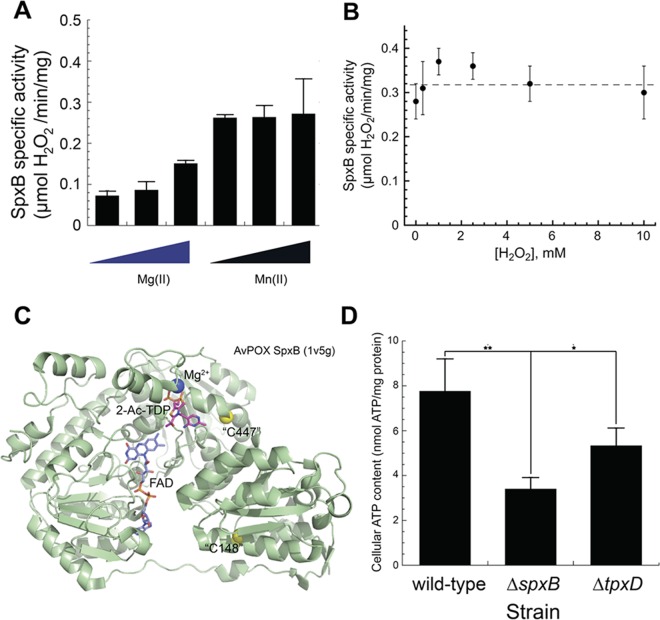
*S. pneumoniae* SpxB activity is resistant to H_2_O_2_ exposure. (A) Specific activity of *S. pneumoniae* SpxB in the presence of 0.5, 1, and 2 mM Mg(II) or Mn(II). (B) Specific activity of *S. pneumoniae* SpxB after incubation with H_2_O_2_ (0 to 10 mM) at pH 7.0. The average activity is 0.32 ± 0.04 µmol·min^−1^·mg^−1^ protein (dashed line) and is independent of H_2_O_2_ over this concentration range. (C) Structure of *Aerococcus viridans* (pyruvate oxidase [AvPOX] subunit [1V5G; 69% identity, 82% similarity to *S. pneumoniae* SpxB]) (94) highlighting the FAD and TPP cofactors, the latter as the 2-acetyl-thiamine diphosphate (2-Ac-TDP) reaction intermediate, the Mg(II) ion, and the approximate position of two Cys (C148 and C447, based on *S. pneumoniae* residue numbering) not found in *A. viridans* pyruvate oxidase. C447 is quite close to the active site, while C148 is sulfonylated in cells (Table S2D). Mg(II) is octahedrally coordinated by D439, N466, D468 O′ (all conserved in the *S. pneumoniae* SpxB), a water molecule, and substrate. (D) Excess proteome sulfenylation in a Δ*tpxD* mutant (Fig. 4) inhibits ATP generation in *S. pneumoniae*. The ATP content (nanomoles of ATP per milligram of protein) of mid-log-phase (OD_620_ ≈ 0.3) cultures in BHI under microaerophilic conditions was measured for the wild-type, Δ*spxB*, and Δ*tpxD* strains. ATP content was found to be significantly lower for both Δ*spxB* (*P* < 0.01) and Δ*tpxD* (*P* < 0.05) strains based on 2-tailed unpaired *t* tests.

We next assessed the role that sulfenylation might play in regulating the activity of SpxB, a major sulfenylation target detected in our profiling experiments (Fig. 3 to 5; Table S2D). Most bacterial pyruvate oxidases require Mg(II) to bind the thiamine pyrophosphate cofactor in the active site (70). Some Mg-containing enzymes are also active with Mn (70, 71), and we find that both Mg and Mn stimulate *S. pneumoniae* SpxB activity, with Mn functioning as the more efficacious cofactor (Fig. 8A). Mn-loaded SpxB activity is unaffected by sulfenylation, since H_2_O_2_ generation remains unaffected when increased H_2_O_2_ is added to the enzyme (Fig. 8B). Given the little impact of H_2_O_2_ on specific activity, it seems possible that SpxB might function as a hydrogen peroxide “sink,” a role consistent with its high cellular abundance (Fig. 5A). This was not investigated further here; however, the structure of a closely related pyruvate oxidase from *Aerococcus viridans* suggests that C475 in *S. pneumoniae* SpxB, likely solvent-exposed Cys and close to the active site, may function as the sulfenylated cysteine in SpxB (Fig. 8C); interestingly, however, C148 is sulfonylated in cells (Table S2D).

### Loss of TpxD impairs ATP production in cells.

The Δ*tpxD* strain exhibits a marked impairment in virulence and resistance to oxidative stress similar to that observed in *spxB* mutants (39). It has been suggested that Δ*spxB* strains exhibit this phenotype due to a reduced ability to generate ATP under conditions of both endogenous and exogenous ROS (7). Similarly, the sulfenylation of glycolytic and pyruvate node enzymes in *S. pneumoniae* (Fig. 6A) suggests that H_2_O_2_ may modulate the flux through glycolysis and therefore ATP production in the absence of TpxD. As previously reported, *ΔspxB* mutants contain substantially lower ATP (≈50%) compared to wild-type cells (Fig. 8D) (7), primarily attributed to lower AckA activity (Fig. 1) (8). We show here that pneumococcal strains lacking *tpxD* also show decreased ATP content (≈30%) compared to wild-type cells (Fig. 8D), suggesting that loss of TpxD significantly impacts ATP synthesis. We suggest that TpxD exerts cellular control of glycolysis or in the pyruvate node directly in *S. pneumoniae*, given that GapA, pyruvate kinase, LctO, and AckA are all sulfenylation targets under these growth conditions (Fig. 5; Table S2A and B).

## DISCUSSION

The findings presented here describe the biological and chemical adaptations of *S. pneumoniae* to endogenous oxidative stress, which occurs as a result of growing aerobically versus anaerobically. It has been known for over a decade that pyruvate oxidase (SpxB) protects *S. pneumoniae* against exogenous H_2_O_2_, even though it biosynthesizes a substantial fraction of the total endogenous H_2_O_2_ (7) (Fig. 2 to 3). The inability of *ΔspxB* strains to limit the depletion of ATP during oxidative stress was proposed to be the primary reason for increased sensitivity to sublethal and lethal H_2_O_2_ stresses (7). In this study, we extend this protective effect to lactate oxidase, LctO, which also generates H_2_O_2_ and, like SpxB, is a direct sulfenylation target (Fig. 5). Previous findings reveal that SpxB is the dominant H_2_O_2_-generating enzyme where deletion mutants showed a 90% reduction in H_2_O_2_ production. Therefore, our finding that *ΔlctO* strains generate only 40% of the H_2_O_2_ (Fig. 2) of wild-type strains and have increased H_2_O_2_ sensitivity (Table S1C) highlights the important role that LctO plays in pneumococcal metabolism. LctO becomes protective by regenerating the pyruvate pool for SpxB activity, thereby allowing for maximal SpxB turnover and cellular ATP generation. Although the specific activity of SpxB is not adversely affected by H_2_O_2_, it is unknown if sulfenylation of LctO and AckA have any impact on enzymatic activity. In addition, we provide genetic evidence in support of a functional pyruvate dehydrogenase (PDHC) complex in *S. pneumoniae* D39 (*spd1025* to *spd1028*), which extends our understanding of the pyruvate node in the generation of acetyl-CoA in *S. pneumoniae* (Fig. 1). The presence of a functional PDHC in the D39 strain is fully consistent with recent studies in the serotype 4 *S. pneumoniae* TIGR4 strain (41). Such a PDHC would allow the organism to generate ATP in the absence of a functional SpxB from PDHC-derived acetyl-CoA (41). Both pathways may well be operative in the wild-type strain under the aerobic growth conditions employed here, with the SpxB pathway the preferred pathway (Fig. 8). The pyruvate formate lyase (PFL) pathway in conjunction with phosphate acetyltransferase (Pta), will be the major pathway under anaerobic conditions.

In other bacteria, dedicated and distinct ROS-sensing repressors function as oxidative stress sensors that respond to these specific acute exogenous stressors (21). However, *S. pneumoniae* does not encode any of these repressors, perhaps due to the continuous exposure of endogenous ROS during aerobic growth. Instead, others, including SpxR, Rgg, RitR, NmlR, PsaR, and CiaRH, have been linked to regulation of gene expression, directly or indirectly, in response to oxidative stress (2), with the molecular details beyond the Mn sensor PsaR (72) largely undefined. SpxR, which senses the energy and metabolic state of pneumococcus, has been identified as a positive regulator of *spxB* and another 20 genes (15). Interestingly, another *spxR* regulon member, *strH*, which encodes an important exoglycosidase implicated in colonization in the airway (46), also shows a large (4-fold) increase in aerobic versus anaerobic growth (15) (Table 3). Among other genes that show differential expression, *tpxD* and *piuB* are regulated by the Rgg transcription regulator (23), and the iron regulator RitR, respectively. In addition, TpxD is involved in the negative regulation of *psaBCA* (39), which is consistent with our findings that *psaBCA* expression decreases while* tpxD* expression increases under aerobic versus anaerobic conditions (Table 3). How *spd0091*, *sodA*, and the sole iron-sulfur (Fe-S) protein biogenesis system in *S. pneumoniae* (*sufCDSUB*) (Table 3) (73–75) are regulated is currently unknown. These results suggest that gene expression control of aerobiosis versus anaerobiosis in *S. pneumoniae* is mediated by multiple characterized and unknown regulators.

The functional roles played by the ROS-resistant Fe-S protein biogenesis system Suf (mobilization of sulfur) and the putative sulfurtransferase SPD_0091 under aerobic conditions are unknown. The SufBCD complex (76) is highly abundant in our cells (Fig. 5A; Table S2A and B) in contrast to the cysteine desulfurase SufS and the putative Fe-S scaffold protein SufU; furthermore, SufB and SufD are sulfenylated or sulfonylated (SufD) in cells (Fig. 5C). Fe-S client proteins in *S. pneumoniae* are not well characterized, and it is interesting to note that *S. pneumoniae* does not conserve the [4Fe-4S] clusters of enzymes required for genome maintenance and repair found in *E. coli* and other organisms (45), including the DNA glycosylase MutY (*spd1086*), Nth exonuclease III (*spd1135*), *dinG* family helicases (*spd0705*), or DNA primase (*spd0957*;* dnaG*). In fact, a bioinformatics search for iron-sulfur proteins in *S. pneumoniae* D39 reveals just 11 strong candidate [4Fe-4S] proteins (77), with seven of these known or predicted radical *S*-adenosylmethionine (SAM) enzymes that function as “activases” to generate stable glycyl/thiyl radicals on substrate proteins (Table S2F) (78). Both the anaerobic RNR (NrdG) and PFL (pyruvate formate lyase) (PflA) are dependent on these enzymes, and we show here that the catalytic subunit of PFL, PflB, is required for anaerobic growth (Table 2). These findings therefore provide an explanation as to why the *suf* genes are essential for anaerobic growth (Table 2); note that in the serotype 4 TIGR4 strain, the *suf* genes are also essential by transposon sequencing (Tn-Seq) analysis (79). In contrast, aerobic targets for [4Fe-4S] clusters made possible by upregulation of the *suf* system under aerobic conditions (Table 3) remain undefined. In this context, it is interesting that the [4Fe-4S]-containing l-serine dehydratase (Table S2D) which deaminates l-Ser to pyruvate and ammonia, potentially provides a source of pyruvate under conditions where glycolytic flux might be compromised. However, these enzymes tend to be oxygen labile (80).

SPD_0091 is highly induced under aerobic conditions, consistent with previous findings (23), and is a direct sulfenylation target in cells (Fig. 5C; Table S2D). SPD_0091 is predicted to be a multidomain protein that harbors a central near-canonical rhodanese domain (RHD), flanked by an N-terminal domain and C-terminal pseudorhodanese domain (an RHD lacking an active-site Cys). Although the structure of SPD_0091 is unknown, *L. pneumophilia* Lpg2838, a homologue of SPD_0091, Fig. S7B), reveals an N-terminal α-β sandwich domain connected to the RHD via a disordered linker. The RHD harbors an active-site Cys (C177) that is disulfide bonded to C182 in the structure, both of which are conserved in pneumococcal SPD_0091. The function of Lpg2398, like SPD_0091, is unknown. Rhodaneses are sulfurtransferases that carry bioactive sulfur as active-site persulfides and function as cellular sulfur donors in Fe-S cluster biogenesis, sulfur assimilation, H_2_S oxidation, and the biosynthesis of sulfur-containing cofactors (81, 82). In addition to their role as sulfur donors, some rhodaneses function in thiyl radical chemistry and as targets of sulfenylation in bacterial cells (70), as observed here. The fact that SPD_0091 harbors a Cys pair, rather a single active-site Cys (Fig. S7B), more strongly suggests a role in thiol-disulfide chemistry or oxidative stress management than as a persulfide carrier. However, SPD_0091 is not required to protect cells against endogenous H_2_O_2_ stress since the Δ*spd0091* strain, like the Δ*sodA* and Δ*tpxD* strains, exhibits no obvious growth phenotypes on either TSA II BA plates or in our BHI broth under aerobic conditions, nor do we observe significant differences in exogenous H_2_O_2_ sensitivity between these deletion strains and the wild-type strains (data not shown).

These transcriptomic changes occur coincidentally with significant proteome sulfenylation derived from endogenous H_2_O_2_ production, the level of which is globally controlled by the thiol peroxidase TpxD. The full physiological adaptation of proteome sulfenylation induced by endogenous H_2_O_2_ is not yet known, but sulfenylation levels clearly impact ATP synthesis, which pinpoints glycolysis, sugar utilization, and capsule biosynthesis as key points of regulation by sulfenylation. The pneumococcal GapA may be more resistant to H_2_O_2_-mediated inhibition relative to non-lactic acid bacterial GAPDH enzymes from *S. aureus* or *P. aeruginosa*, which leads to stalling of glycolysis *in vivo* (51, 83); however, pneumococcal GapA conserves all key elements known to control H_2_O_2_ reactivity (84). Metabolic and transcriptomic analyses of *S. aureus* and *P. aeruginosa* cultures under chronic exogenous H_2_O_2_ stress (3 to 7 mM) show a significant metabolic rerouting toward the pentose phosphate pathway (PPP) in order to regenerate the cellular reductant NAPDH for ROS detoxification systems, including thiol and glutathione peroxidases (51, 85). Interestingly, the fate of the product of the oxidative phase of the PPP, ribulose-5-phosphate, may also be subject to regulation by sulfenylation (Fig. 6B).

Although TpxD is the master regulator of endogenous proteome sulfenylation, these levels can also be influenced by changes in transition metal availability, but in distinct ways. Mn stress, in particular, increases proteome sulfenylation by ~50% (Fig. S2), an effect traced to dysregulation of the bioavailable Fe and resultant changes in the Fe/Mn ratio (86, 87). In group A streptococci (GAS), Mn toxicity sensitizes the bacteria to neutrophil-mediated killing and H_2_O_2_ stress (88). This sensitivity is also tied to changes in the intracellular Mn/Fe ratio leading to Mn-mediated PerR repression and thus altered regulation of the oxidative stress response. However, instead of an inducible transcriptomic response to alterations in the Mn/Fe ratio via PerR, *S. pneumoniae* employs a chemical adaptation strategy to modulate the impact of endogenous H_2_O_2_ production on cell metabolism. Part of this adaptation is “self-sulfenylation” of SpxB, which although catalytically silent (Fig. 8C), may allow SpxB to function as an H_2_O_2_ “sink.” Although not tested here, this hypothesis is consistent with the fact that Δ*spxB* strains are more sensitive to exogenous H_2_O_2_.

We propose that *S. pneumoniae* exploits endogenous H_2_O_2_ to function as an intracellular signaling molecule that modulates glycolytic flux (84), pyruvate metabolism, nucleotide biosynthesis, and capsule biosynthesis via protein sulfenylation. Indeed, chemical adaptation to aerobic growth is a critical aspect in the virulence of *S. pneumoniae*, particularly during the colonization phase; furthermore, *spxB* mutants lead to increased capsule production as well as altered sugar utilization (16). Regulation of capsule formation is an important part of pneumococcal evasion of the host immune response, particular during phagocytosis (89) and perhaps during sepsis, i.e., as the local microenvironment becomes more anaerobic. Additionally, hypervirulent serotype 1 pneumococcal strains have been found to harbor *spxB* mutations resulting in little to no H_2_O_2_ production; as expected from this H_2_O_2_ signaling model, these mutants are impaired in colonization relative to wild-type strains (18). The extent to which these features characterize other pneumococcal strains is unknown, since serotype 2 Δ*spxB* strains are less virulent. Studies are under way to integrate recently developed quantitative chemoproteomics strategies (61, 90) to map sites of proteome sulfenylation and quantify fractional sulfenylation levels with a targeted metabolomics analyses, to better elucidate the impact of endogenous H_2_O_2_ versus exogenous immune system-derived ROS stress on pneumococcal physiology.

## MATERIALS AND METHODS

### Chemicals and reagents.

All water used in these experiments was Milli-Q deionized (>18 MΩ), and the buffers were obtained from Fisher Scientific. 5,5-Dimethyl–1,3-cyclohexanedione (dimedone) was obtained from Sigma-Aldrich, and the solid was dissolved in a 1:1 solution of dimethyl sulfoxide (DMSO) and 500 mM Bis-Tris (pH 7.4). All antibiotics, desferrioxamine, ferric chloride, manganese(II), chloride tetrahydrate, nitrilotriacetic acid (NTA), and reduced glutathione were purchased from Sigma-Aldrich; zinc sulfate was obtained from Alfa Aesar. Daz-2 dimedone was obtained from Caymen Chemicals and dissolved in DMSO. Dithiothreitol (DTT) was obtained from Sigma and dissolved in Milli-Q water. All other reagents were obtained as indicated below. An Ätka 10 purifier (GE) was used for all chromatographic steps.

### Bacterial strains and growth conditions.

Detailed genotypes and descriptions of serotype 2 *Streptococcus pneumoniae* strain D39 and its derivative strains used in this study are listed in Table S1A and in Text S1 in the supplemental material. Cultures were grown statically in brain heart infusion broth (BHI) with limited aeration or on plates containing modified Trypticase soy agar II (Becton, Dickinson; BD) and 5% (vol/vol) defibrinated sheep blood (Remel) (TSAII BA) lacking antibiotics at 37°C. We refer to growth with limited aeration as aerobic growth in this study. For cultures grown under this condition, 5 ml of cultures was incubated in 16- by 100-mm glass tubes in an atmosphere of 5% CO_2_ in loosely capped tubes, which were gently inverted three times before the OD_620_ was measured with a Spectronic 20 spectrophotometer fitted for measurement of capped tubes (outer diameter, 16 mm). For growth experiments, bacteria were inoculated into BHI broth from frozen cultures or colonies, serially diluted into the same medium, and propagated overnight for 15 to 18 h. Overnight cultures that were still in exponential phase (OD_620_ = 0.1 to 0.4) were diluted back to an OD_620_ of ≈0.005 to start final cultures, which lacked antibiotics. All anaerobic procedures were carried out in a Coy anaerobic chamber that maintains an atmosphere of 2.0% hydrogen, 7% CO_2_, and 91% nitrogen. BHI and TSAII BA plates used for anaerobic experiments were equilibrated overnight in this atmosphere.

10.1128/mSphere.00291-16.10 TEXT S1  Supplemental methods. Download Text S1, PDF file, 0.1 MB. Copyright © 2017 Lisher et al. 2017 Lisher et al. This content is distributed under the terms of the Creative Commons Attribution 4.0 International license.

### Transposon mutagenesis and inverse PCR.

The *lctO*::Mariner mutant in the R6 genetic background was isolated during an extension of the genetic screen performed for a previous study (15) (see Results). Transposon mutagenesis and inverse PCR procedures were performed as previously described (15). Primers used for sequencing of the *lctO* region to identify the location of the transposon are listed in Table S1B.

### Transformation assays with ΔPDHC and Δ*spxB*.

ΔPDHC::P_c_-(*kan-rpsL*^*+*^) and Δ*spxB*::P_c_-*erm* amplicons and positive-control Δ*pbp1b*::P_c_-(*kan-rpsL*^*+*^) and Δ*pbp1b*::P_c_-*erm* amplicons with ~1-kb flanking DNA sequences were obtained from PCRs using primers and templates listed in Table 1. *pbp1b*, which codes for penicillin binding protein 1B, is not essential and is not involved in oxidative stress in pneumococcus. The transformation assay was performed as reported in reference 91, except for the use of 200 µl of recipient strains grown to an OD_620_ of ≈0.05 and 100 ng of purified PCR amplicons.

### Microarray analysis.

Three independent hybridizations for microarray analysis using two independent sets of RNA preparations from *Streptococcus pneumoniae* strain IU1690 (D39) and one RNA set from strain IU1781 (D39 *rpsL1*) were performed. For cultures grown under the limited-aeration condition, bacterial strains were grown statically in BHI medium (Bacto BHI; Becton, Dickinson) at 37°C in an atmosphere of 5% CO_2_ and 95% air overnight. Overnight (~15-h) limited-aeration cultures that were in log phase (OD_620_ of ~0.1 to 0.3) were diluted to an OD_620_ of ~0.005 in 25 ml of BHI medium in 50-ml conical tubes with loose caps and grown at 37°C and an atmosphere of 5% CO_2_. To prepare anaerobic medium, 200 ml of BHI medium in a 250-ml glass bottle with loose cap was equilibrated in the Coy anaerobic chamber for 15 h. The same overnight limited-aeration culture was similarly diluted into equilibrated anaerobic medium and incubated at 37°C in the Coy anaerobic chamber. Total RNAs from both limited-aeration and anaerobic cultures were extracted from exponentially growing cultures (OD_620_ ~ 0.2) using a hot lysis/acid phenol procedure followed by on-column DNase treatment and purification using the RNeasy minikit (Qiagen) as described in reference 15. Cultures grown anaerobically to an OD_620_ of ~0.2 were removed from the anaerobic hood and added immediately (less than 10 s in aerobic condition) to boiling lysis buffer.

*S. pneumoniae* microarrays (Ocimum Biosolutions) covering 2,018 open reading frames (ORFs) of the R6 genome, which lacks *cps2B* to *cps2G* of D39 genome, were used. Synthesis, labeling, and hybridization to *S. pneumoniae* microarrays (Ocimum Biosolutions), scanning, and analysis using the Cyber-T web interface were performed as described previously (15). Data were normalized without background subtraction by the global LOWESS method using BASE (BioArray Software Environment; http://base.thep.lu.se/), excluding empty wells and *Arabidopsis thaliana* control spots.

### **Construction of** *E. coli*** overexpression plasmids and protein purification.**

The genes of interest were amplified from *S. pneumoniae* D39 genomic DNA using cloning primers with *Bam*HI and *Nde*I restriction sites. The locus tag designations for GapA, SpxB, TpxD, TrxA, and TrxB are *spd1823*, *spd0636*, *spd1464*, *spd1567*, and *spd1287*, respectively. These inserts were cloned into the expression vector pHis-parallel under transcriptional control of the T7 promoter (92), with the integrity of all plasmids verified by sequencing. The expression plasmids were transformed into competent Rosetta BL21(DE3)/pLysS cells, plated onto a plate containing ampicillin and chloramphenicol (100 µg/ml and 37 µg/ml, respectively), and grown overnight at 37°C. Single colonies were used to inoculate 100-ml LB cultures containing both ampicillin and chloramphenicol and were grown overnight at 37°C with shaking. The overnight cultures were diluted into 1 liter LB and grown at 37°C with shaking. Overexpression was accomplished by induction of 1 liter of mid-log LB cultures with 0.4 mM IPTG (isopropyl-β-d-thiogalactopyranoside [INALCO]) for 2.5 h at 37°C. Cells were harvested and resuspended in 25 mM Tris (pH 8.0), 300 mM NaCl, 3 mM TCEP [tris(2-carboxyethyl)phosphine], and 25 mM imidazole. The resuspended cells were lysed by sonication and centrifuged at 15,500 × *g* for 20 min. The lysate was purified using HisTrap FF columns (GE Healthcare) with a step gradient from 25 mM to 500 mM imidazole. Fractions containing the desired protein were determined by SDS-PAGE gels and pooled for further chromatography. The pooled samples were concentrated using centrifugal filter units (Millipore) and applied to a size exclusion column (Superdex 200 prep grade or Superdex 75 prep grade). The fractions were collected and dialyzed against a mixture of Chelexed 25 mM Tris (pH 8.0), 300 mM NaCl, and 3 mM DTT, aliquoted, and stored at −80°C until use. Proteins were identified by SDS-PAGE and confirmed by electrospray ionization-mass spectrometry (ESI-MS) for purity and mass. Protein concentrations were calculated using the predicted extinction coefficients of the His-tagged construct at 280 nm (ProtParam).

### Western blotting of sulfenylated proteins.

Whole-cell lysates were prepared using the FastPrep method. Briefly, strains of *S. pneumoniae* were grown overnight in BHI from ice stocks and then diluted to an OD_620_ of ~0.004 in 20 ml BHI in a 50-ml loosely capped conical tube and were allowed to grow in an atmosphere of 5% CO_2_ at 37°C to an optical density of ≈0.1 when either 10 mM dimedone or 1 mM Daz-2 was added. For strains with metal stresses, strains were diluted to an OD_620_ of ≈0.004 in 20 ml BHI containing the indicated added concentration of Zn (0.2 mM), Cu(II) (0.5 mM), Fe(III) (0.05 mM), and Mn (0.1 mM). For strains incubated with DFO, the final concentration was 15 µM. Cells were harvested by centrifugation (10,000 × *g* for 10 min) after 1 h, supernatants were discarded, and pellets were placed on ice and suspended in a cold mixture of 1.0 ml 20 mM Tris (pH 7.4), 5 mM iodoacetamide, and 8 μl of protease inhibitor cocktail set III (Calbiochem) and transferred to chilled Lysing matrix B tubes (MP Biomedicals). Matrix tubes were secured in a 24- by 2-ml tube adaptor in a FastPrep-24 instrument (MP Biomedicals) stored at 4°C. Cells were disrupted by five runs of 40 s each at a speed setting of 6.0 m/s where the first three runs were consecutive, the samples were cooled for 5 min, and the last two runs were also consecutive. Lysed cell mixtures were placed on ice and centrifuged at 10,000 × *g* for 1 to 5 min at 4°C. One hundred microliters of supernatant was transferred to a tube containing 100 μl of 2× Laemmli sample buffer (containing 5% [vol/vol] of freshly added β-mercaptoethanol), boiled for 5 min, and placed on ice. Protein content (milligrams per milliliter) was determined using the DC protein assay (Bio-Rad). Individual gel lanes were loaded with 10 µg total protein, with visualization and relative quantification of sulfenylated proteins achieved by Western blotting with primary anti-cysteine sulfenic acid polyclonal antibody (EMD-Millipore, 07-2139 [1:1,000 dilution]) and an IVIS *in vivo* imaging system (PerkinElmer).

### Peptide identification by mass spectrometry.

Experiments were performed on either an LTQ Velos linear ion trap or a hybrid LTQ Orbitrap XL (Thermo Fischer Scientific) coupled to Eksigent nano-high-performance liquid chromatography (nano-HPLC) systems (Waters, Milford, MA). For samples analyzed on the LTQ Velos, peptides were separated on an in-house packed reverse-phase C_18_ column with a 60-min gradient elution, with principal elution occurring with a gradient from 8% B to 33% B from 1 to 51 min. Buffer A consisted of 2% acetonitrile and 0.1% formic acid in water, while buffer B consisted of 0.1% formic acid in acetonitrile. The Velos was configured to acquire a survey scan over the mass range 300 to 1,600 *m*/*z*. This was followed by MS/MS on the top 8 most intense precursor ions above a threshold of 1,000 counts. For samples analyzed on the LTQ Orbitrap XL, peptides were separated on an in-house packed reverse-phase C_18_ column with a 60-min gradient elution, with the principal elution occurring with a gradient from 6% B to 32% B from 1 to 49 min. Buffer A consisted of 0.1% formic acid in water, while buffer B consisted of 0.1% formic acid in acetonitrile. The Orbitrap was configured to acquire a survey scan over the mass range 300 to 2,000 *m*/*z* at a resolution of 30,000. This was followed by MS/MS on the top 58 most intense precursor ions above a threshold of 1,000 counts.

### Enrichment and identification of sulfenylated proteins.

Whole-cell lysates were prepared using the FastPrep method as described above with the Daz-2-labeled supernatant stored frozen at −80°C until workup. Seven hundred micrograms of total protein of Daz-2-labeled or control lysate was conjugated to an alkyne-derivatized biotin using Cu(I)-catalyzed azide-alkyne cycloaddition (61). The reaction solution contained 100 µM yn-ACL biotin tag, 2.5 mM ascorbate, 250 µM CuCl_2_, and 500 µM BTTP (93) and was allowed to react for 2 h at room temperature. The reaction was quenched with 5 mM EDTA for 5 min, and samples were buffer exchanged five times into a degassed mixture of 50 mM HEPES and 0.2 mM NaCl (pH 7.0) utilizing Amicon 0.5-ml centrifugal filters (Millipore [3-kDa molecular mass cutoff]) as per the manufacturer’s instructions. The samples was adsorbed onto NeutrAvidin beads (100-µl slurry) prewashed with HEPES buffer, and incubated at room temperature for 1 h with mixing. The beads were washed with 25 mM NH_4_HCO_3_–10% acetonitrile–2 M NaCl twice (500 µl for 5 min) and 25 mM NH_4_HCO_3_–10% acetonitrile twice (500 µl for 5 min) prior to elution. Proteins were eluted in 50 µl 3× Laemmli buffer (final concentration, 1× Laemmli buffer) at 95°C for 15 min. Samples were separated on an SDS-PAGE gel (10% acrylamide) for 1 h at 150 V.

Proteins were stained using the Pierce silver stain kit (Thermo Fisher Scientific) as per the manufacturer’s instructions. Eluted proteins were excised from the gel, destained utilizing the kit reagents, and dehydrated in a centrifugal evaporator for 1 h. Gel pieces were resuspended in 40 µl of 10 mM NH_4_HCO_3_ containing 400 ng proteomics-grade trypsin (Sigma) and digested overnight at 37°C. Samples were quenched with 100 µl 50% acetonitrile–5% formic acid solution. The samples were vortexed and shaken for 20 min to extract the peptides. The process was repeated, and the digest samples were dried completely in a centrifugal evaporator. The peptide samples were resuspended in 25 µl 0.1% formic acid and analyzed by the LTQ Velos as described above. Peptides were identified by searching against the *S. pneumoniae* D39 proteome using Protein Prospector (see Table S2A and B for additional details on data analysis).

### **Determination of** *S. pneumoniae*** GapA and** *S. pneumoniae*** SpxB activity.**

All enzymes were reduced prior to activity assays by incubation with 10 mM DTT for 1 h at room temperature, followed by buffer exchange into a degassed mixture of 50 mM HEPES, 0.2 mM NaCl, and 1 mM EDTA to remove the DTT. The *S. pneumoniae* GapA activity was measured at 25°C spectrophotometrically by measuring the reduction of NAD^+^ at 340 nm. Briefly, enzyme (115 to 150 nM) was mixed with 200 µl assay buffer (50 mM Tris, 15 mM Na_2_HAsO_4_, 0.3 mM d,l-glyceraldehyde-3-phosphate, 0.4 mM NAD^+^) in a 96-well plate in triplicate, and NADH production was monitored with a Synergy H1 multimode plate reader (BioTek) for 3 min. The specific activity was calculated by measuring the initial velocity (Δ*A*_340_ per minute) and converting it to units per milliliter of enzyme from units per milliliter: (Δ*A*_340_/min × *V*_total_)/(6.22 × *V*_enzyme_), where *V*_total_ is the total volume of the reaction in milliliters and *V*_enzyme_ is the volume of enzyme added in milliliters, with a 6.22 mM^−1^ cm^−1^ extinction coefficient used for β-NADH at 340 nm. Determination of units per milligram of enzyme was accomplished by dividing by the concentration of *S. pneumoniae* GapA (milligrams per milliliter) used.

*S. pneumoniae* SpxB activity was measured at 37°C by spectrophotometrically following the generation of a quinoneimine dye at 550 nm through the coupled oxidation of 4-aminoantipyrine and EHPST by horseradish peroxidase (58). Briefly, enzyme (310 to 330 nM) was mixed with 200 µl assay buffer (50 mM KH_2_PO_4_ [pH 6], 0.2 mM flavin adenine dinucleotide [FAD], 0.2 mM TPP, 0.5 to 2 mM divalent metal, 0.48 mM 4-aminoantipyrine, 0.58 mM EHPST, 5 U/ml horseradish peroxidase), and incubated at 37°C for 3 min. Quinoneimine dye formation was monitored in triplicate with the addition of sodium pyruvate to the samples (0.2 mM final concentration) with a Synergy H1 multimode plate reader (BioTek) for 5 min. The specific activity was calculated by measuring the initial velocity (Δ*A*_550_/min) and converting it to units per milliliter of enzyme from units per milliliter by (Δ*A*_550_/min × *V*_total_)/(36.88 × 0.5 × *V*_enzyme_), where *V*_total_ is the total volume of the reaction in milliliters and *V*_enzyme_ is the volume of enzyme added in milliliters, 36.88 is the millimolar extinction coefficient of quinoneimine dye at 550 nm (per millimolar concentration per centimeter), and 0.5 is used to account for the 2 equivalents of H_2_O_2_ consumed to form the dye. The concentration in units per milligram of enzyme was determined by dividing by the concentration of *S. pneumoniae* SpxB (milligrams per milliliter).

### Identification of the repair systems for sulfenylated *S. pneumoniae* GapA.

To test the ability of proteins and low-molecular-weight thiols to repair sulfenylated *S. pneumoniae* GapA, the *S. pneumoniae* GapA activity was measured as described above after incubation with putative repair systems. Prior to incubation with TpxD-TrxA-TrxB, the NADPH-dependent peroxidase activity of the system was tested, adapted from Hajaj et al. (39). Briefly, TpxD (2.5 µM) was incubated with a solution of TrxA (100 µM) and TrxB (50 µM) in reaction buffer (25 mM HEPES, 0.2 M NaCl, 1 mM EDTA [pH 7.0]). Upon addition of 1 mM H_2_O_2_, the decrease in the NADPH absorbance at 340 nm was monitored on Synergy H1 multimode plate reader (BioTek) for 3 min. The repair ability was tested by utilizing freshly sulfenylated *S. pneumoniae* GapA incubated with 2 mM H_2_O_2_ for 5 min. A solution of TpxD-TrxA-TrxB (final concentration, 2.5 µM TpxD, 100 µM TrxA, 50 µM TrxB) was added to the sulfenylated *S. pneumoniae* GapA solution, and NAPDH (final concentration, 0.2 mM) was added to initiate the peroxidase activity for 5 min at room temperature. For low-molecular-weight thiols, 50 mM thiol (glutathione or l-cysteine) was added to freshly sulfenylated *S. pneumoniae* GapA, and the mixture was incubated for 5 min at room temperature. The *S. pneumoniae* GapA activity was measured as described above, and repair was compared against the reductant DTT.

### *In vitro S*-glutathionylation of *S. pneumoniae* GapA.

*S. pneumoniae* GapA was reduced prior to reaction with glutathione by incubation with 10 mM for 1 h at room temperature, followed by buffer exchange into a mixture of degassed 50 mM HEPES, 0.2 mM NaCl, and 1 mM EDTA to remove the DTT. Reduced *S. pneumoniae* GapA (~15 µM) was reacted with or without 2 mM H_2_O_2_ for 5 min, followed by addition of 20 mM reduced glutathione for 10 min at room temperature. The three protein samples were precipitated in 20% trichloroacetic acid (TCA) for 30 min on ice and centrifuged at 13,200 × *g* for 10 min at 4°C. The protein pellets were washed with 200 µl ice-cold acetone and centrifuged twice (13,200 × *g* for 10 min at 4°C) to remove any remaining small molecules. The protein pellet was resuspended in 20 µl Milli-Q water and dried on a centrifugal evaporator. Samples were resuspended in a mixture of 25 mM NH_4_HCO_3_, 10% acetonitrile, and 2 M urea (50 µl) and incubated with 20 mM iodoacetamide for 1 h in the dark to cap reduced thiols prior to digest. Four hundred nanograms of proteomics-grade trypsin from porcine pancreas (Sigma) was added to each sample and digested for 3 h at 37°C. The reaction was quenched with the addition of 10% trifluoroacetic acid (TFA) (final concentration, 0.1%) to the solution. The quenched samples analyzed by the LTQ Orbitrap as described above. Peptides were identified by searching against the His_6_-GapA construct on a Protein Prospector.

### Measurement of cellular ATP content.

*S. pneumoniae* cells were grown as previously described to an OD_620_ of ~0.3. Aliquots of culture (1 ml each) were removed, and cells were harvested at 11,000 × *g* for 10 min at 4°C. Aliquots were washed with 1× phosphate-buffered saline (PBS) and centrifuged again. One aliquot was analyzed for protein content. The other aliquot was resuspended in 750 µl double-distilled water (ddH_2_O) and lysed at 100°C for 10 min. The samples were cooled for 1 min on ice, and two 10-µl aliquots were used to measure ATP content in a 96-well microtiter plate. ATP content (picomoles) was measured by luciferase luminescence utilizing an ATP determination kit (Thermo Fischer Scientific) and a standard curve from 0 to 7.5 pmol ATP. ATP amounts were normalized to total cellular protein content (milligrams) as determined by the DC protein assay (Bio-Rad), and ATP content was reported in nanomoles of ATP per milligram of protein.

### Accession number(s).

Intensity and expression ratio data for all transcripts have been deposited in the GEO database (GenBank accession no. GSE19791).
