# Supplementary figures and images for: Biological and Chemical Adaptation to Endogenous Hydrogen Peroxide Production in Streptococcus pneumoniae D39
Source: mSphere. 2017 Jan 4;2(1):e00291-16. doi: 10.1128/mSphere.00291-16 (PMC5214746; doi:10.1128/mSphere.00291-16)

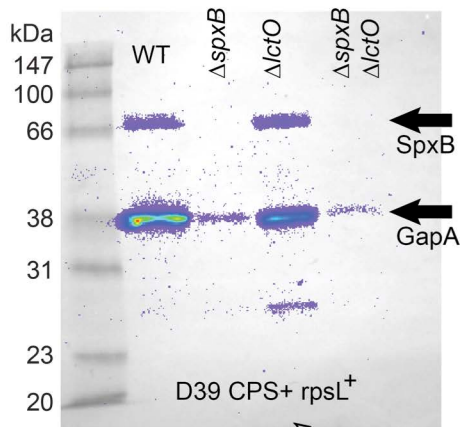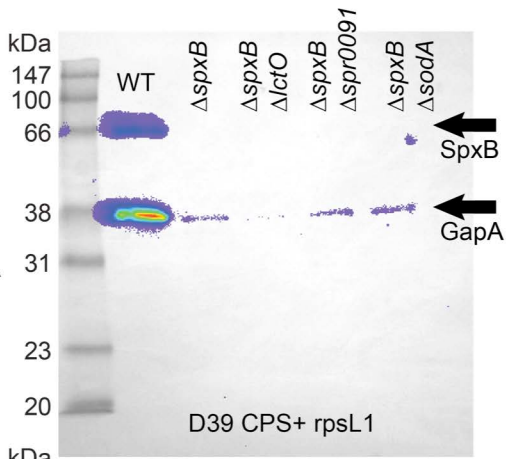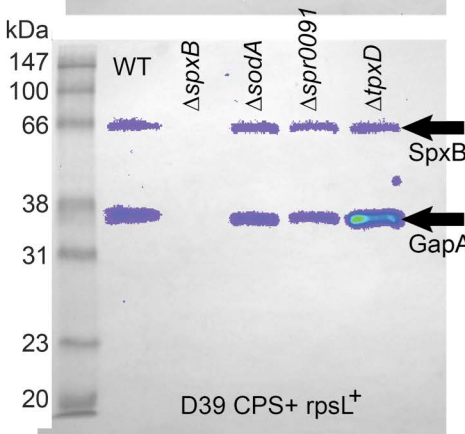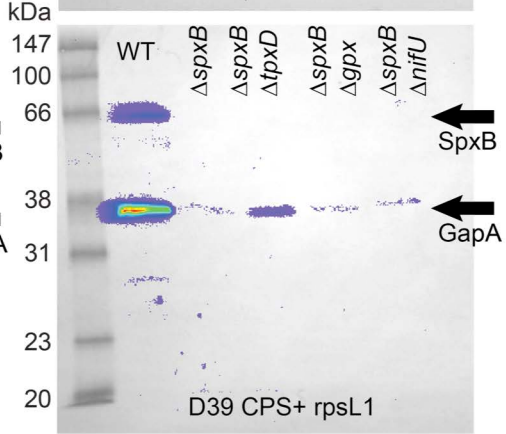

Supplement: FIG S1 [file sph006162217sf2.pdf]

**A**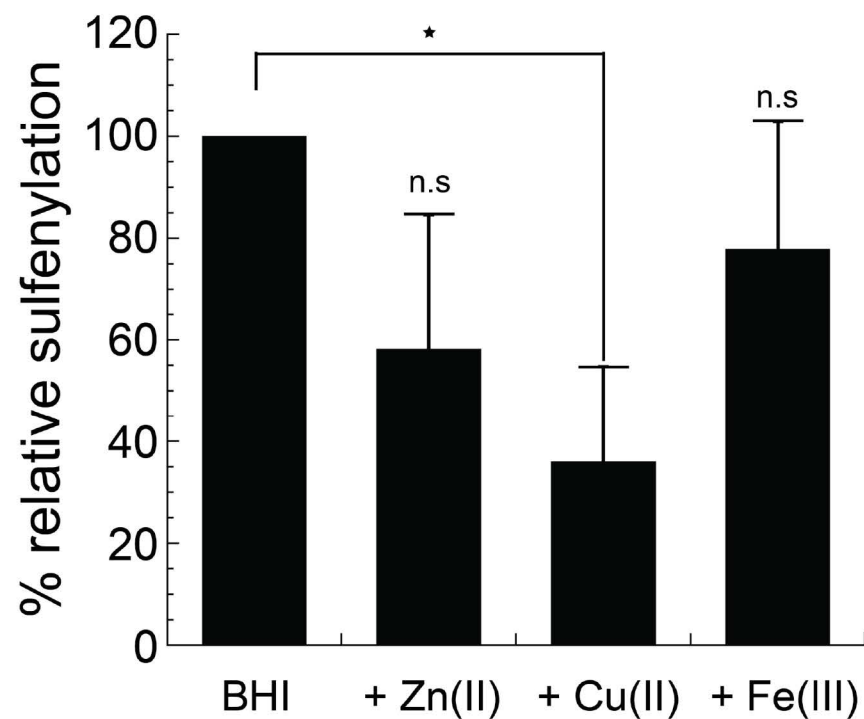**B**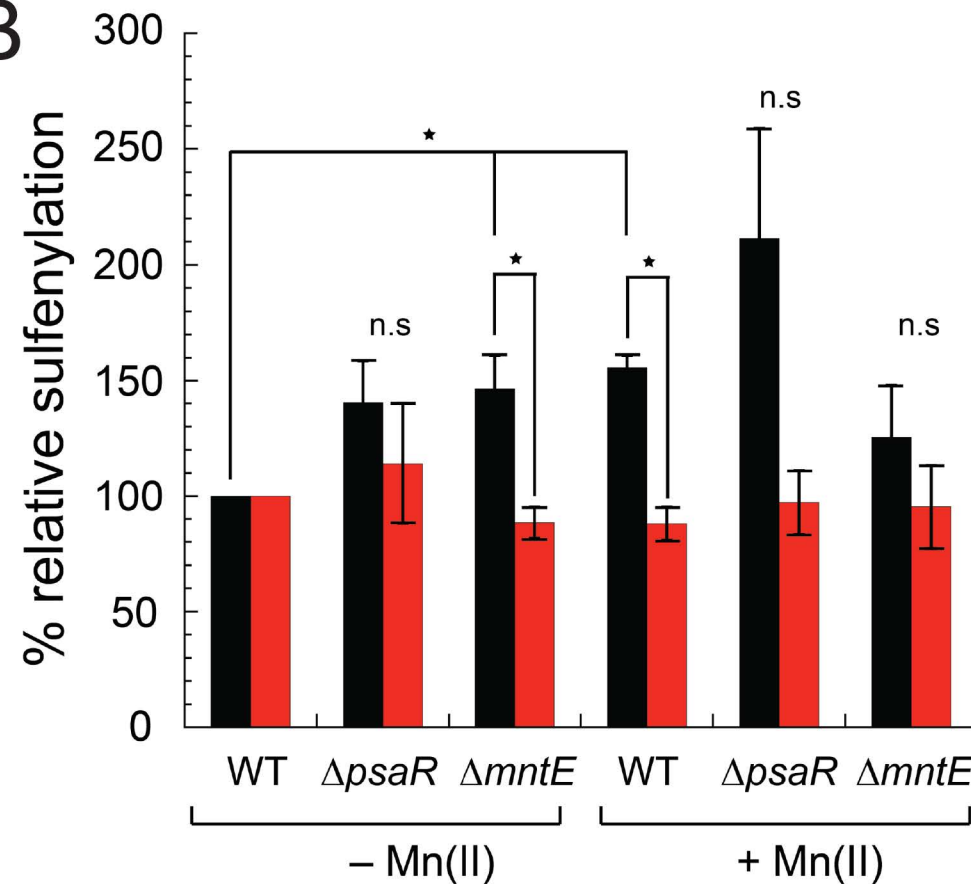**C**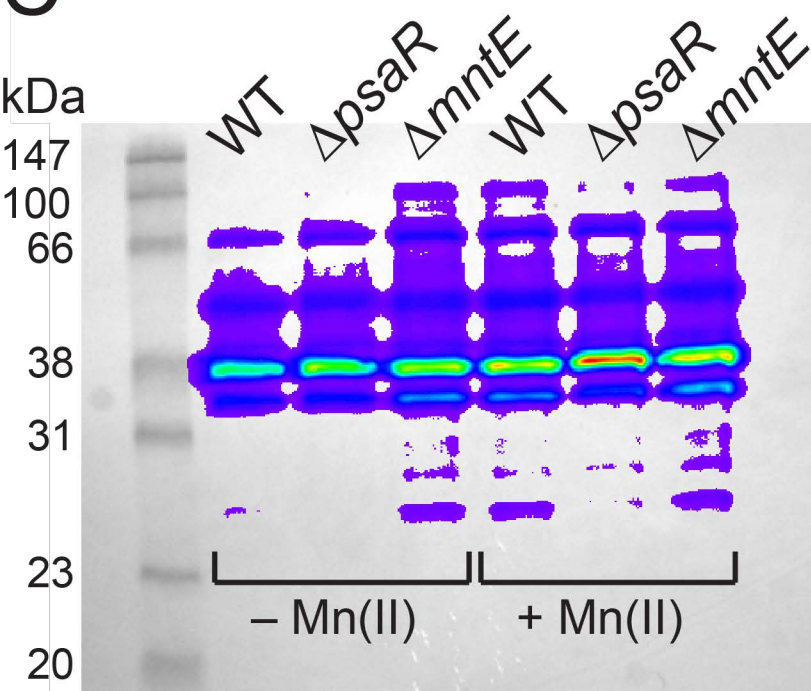**D**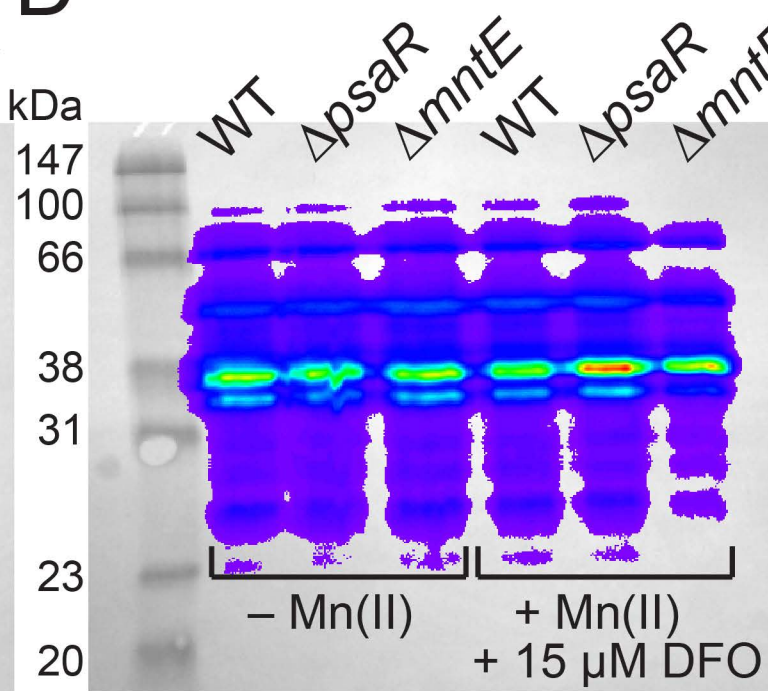**E**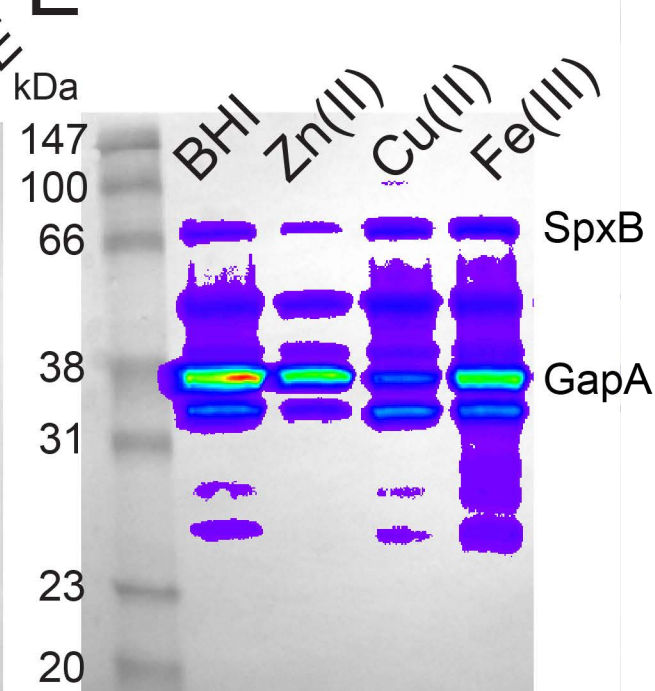

Supplement: FIG S2 [file sph006162217sf3.pdf]

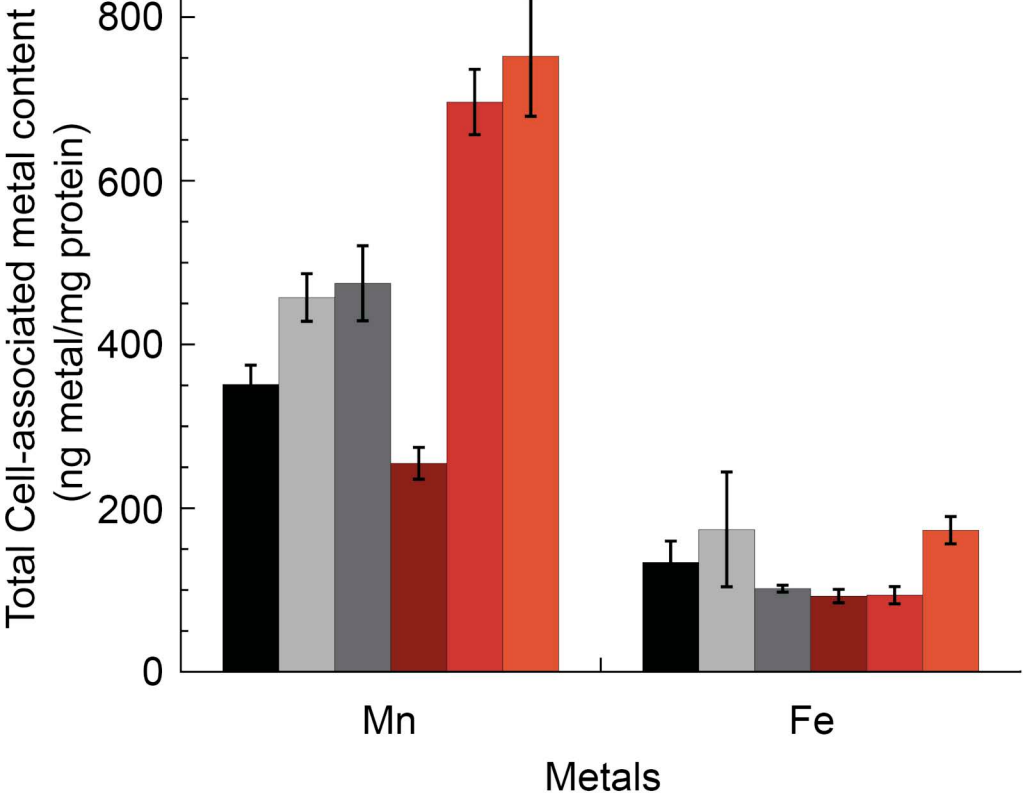

Supplement: FIG S3 [file sph006162217sf4.pdf]

**A**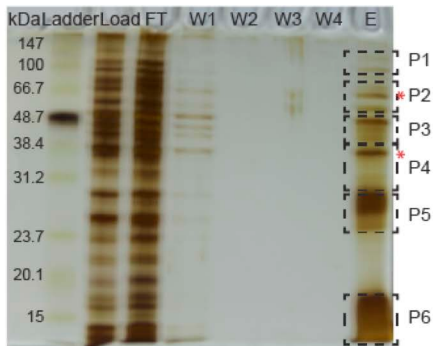**B**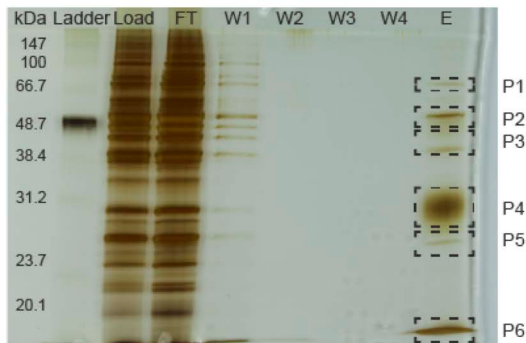**C**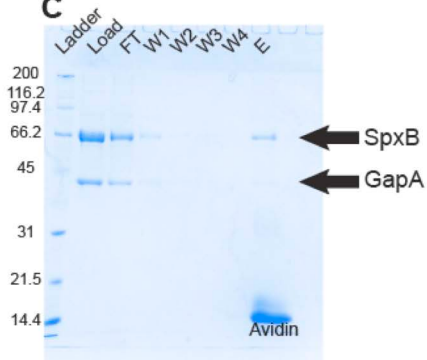

Supplement: FIG S4 [file sph006162217sf5.pdf]

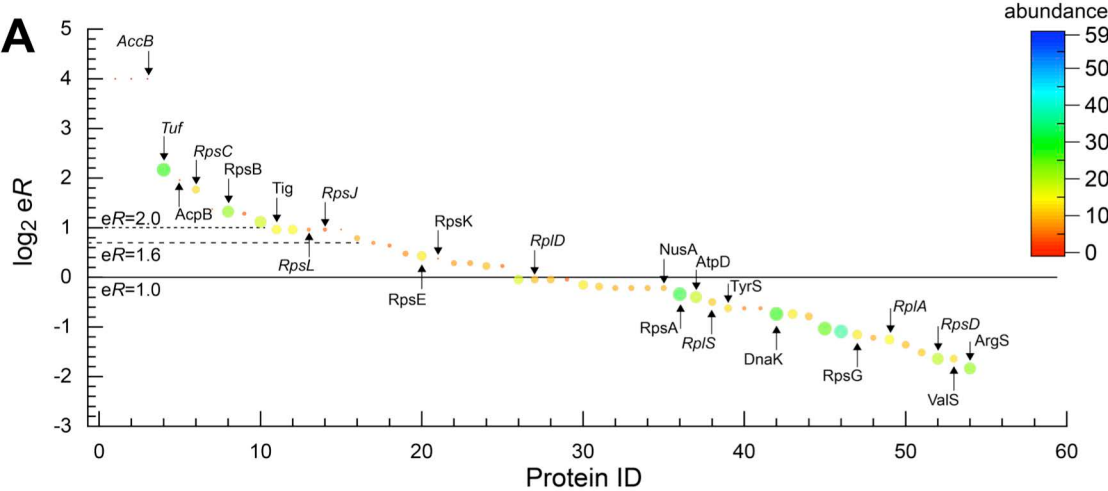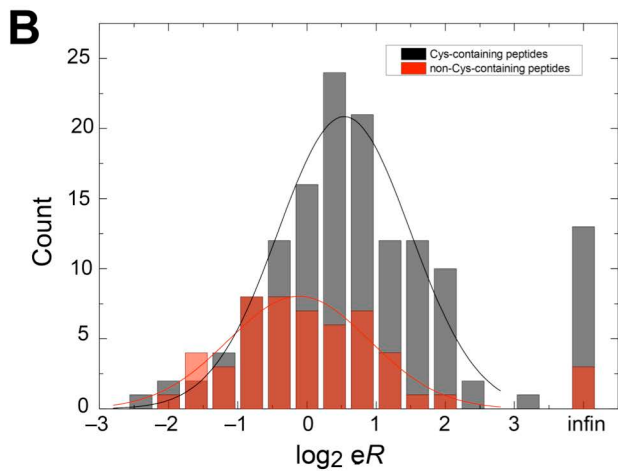

Supplement: FIG S5 [file sph006162217sf6.pdf]

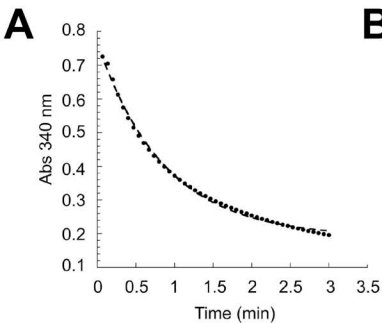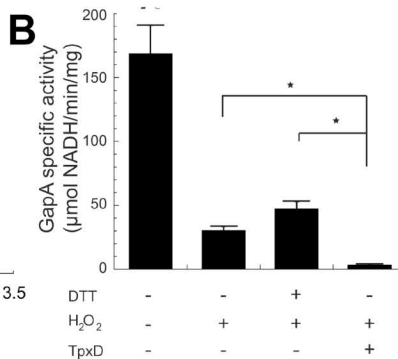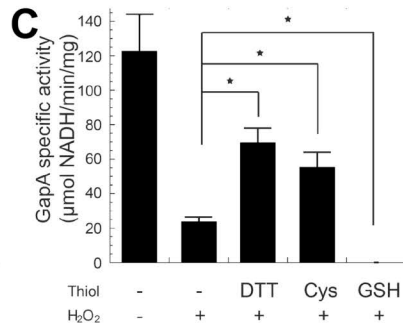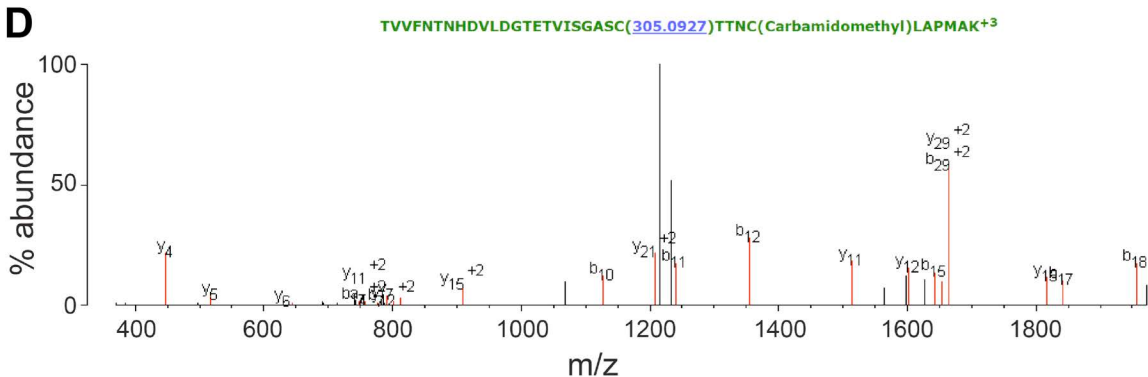

Supplement: FIG S6 [file sph006162217sf7.pdf]

A

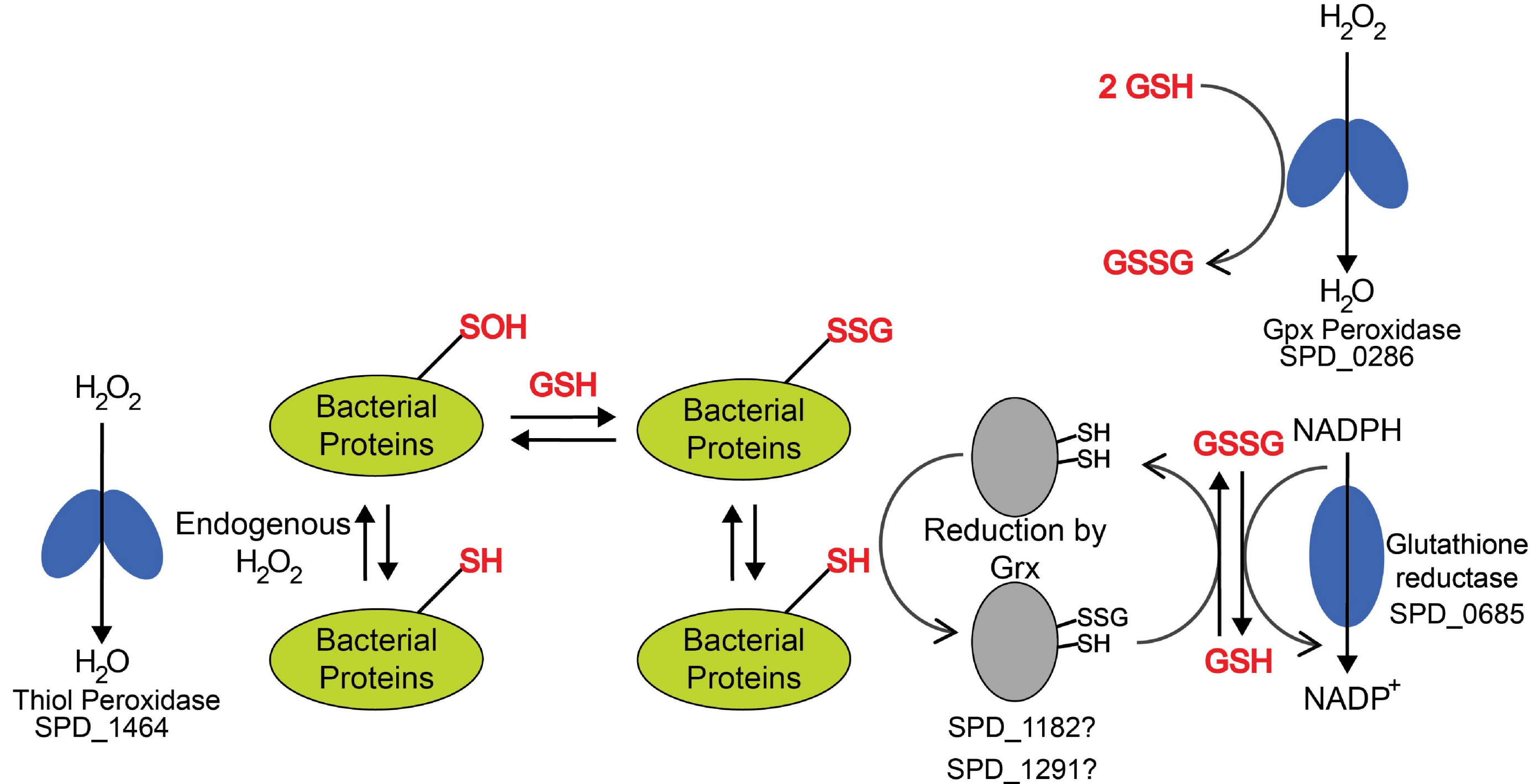

B

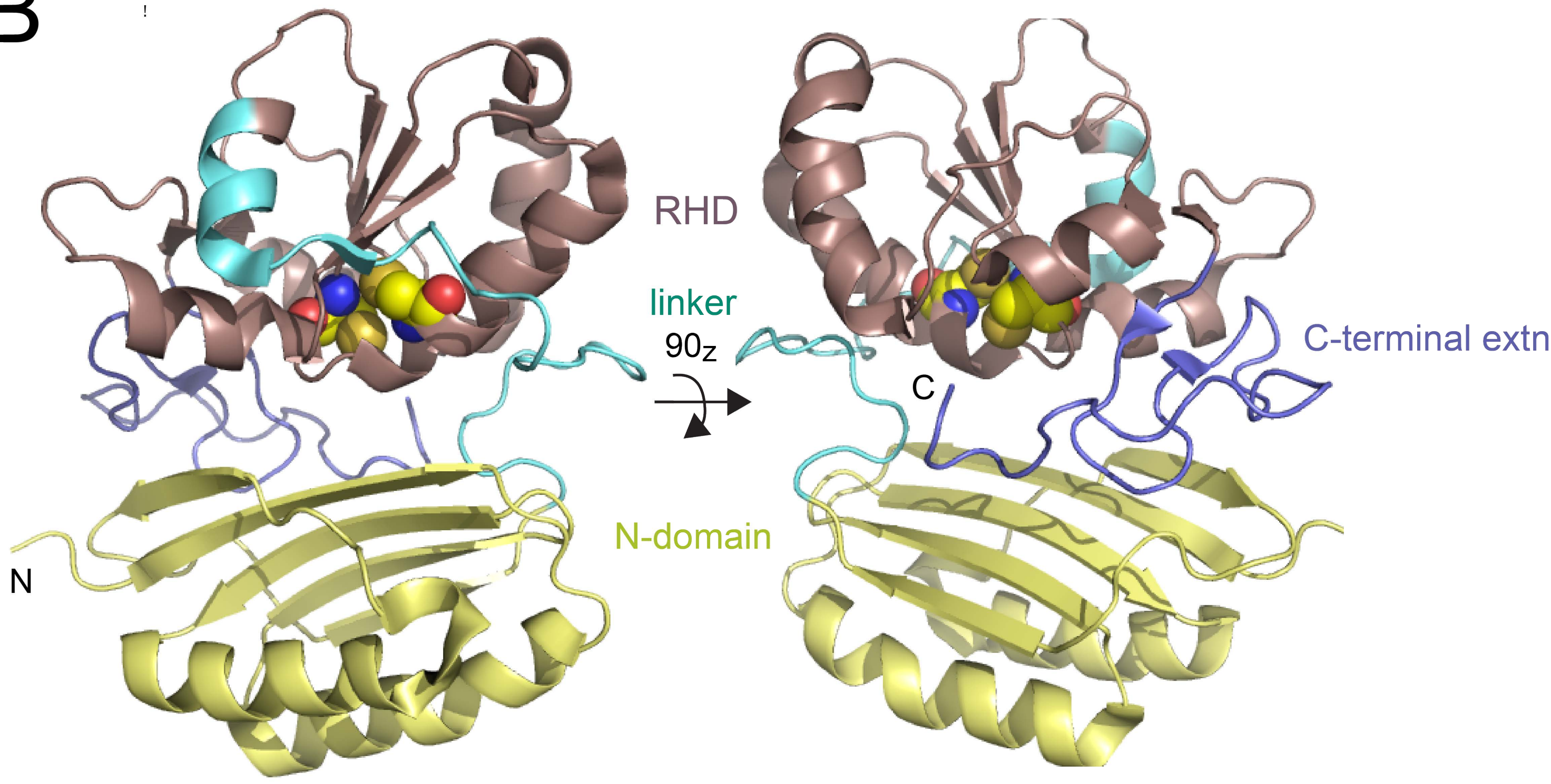

Supplement: FIG S7 [file sph006162217sf8.pdf]
