# Supplementary material for: Biological and Chemical Adaptation to Endogenous Hydrogen Peroxide Production in Streptococcus pneumoniae D39
Source: mSphere. 2017 Jan 4;2(1):e00291-16. doi: 10.1128/mSphere.00291-16 (PMC5214746; doi:10.1128/mSphere.00291-16)
Supplement: TEXT S1 [file sph006162217s1.pdf]

Supplemental Methods

Biological and chemical adaptation to endogenous hydrogen peroxide production in *Streptococcus pneumoniae* D39

Lisher *et al.*

Supplemental Methods

Bacterial strains. Strains containing antibiotic markers were constructed by transforming linear DNA amplicons synthesized by overlapping fusion PCR containing ~ 1kb flanking sequences into CSP-1-induced competent pneumococcal cells as described previously (1). Primers used for the generation of amplicons are listed in Table S2. The amplicon used for construction of IU2633 (D39 *lctO*::Mariner) was amplified using primers RK10 and RK17 (Table S2) from the original transposon mutant IU2674. Strains containing markerless deletion alleles of *spd1025-spd1028 and psaR* in native chromosomal loci (see Table S1) were generated using allele replacement with the P_c-[*kan-rpsL*$^+$] (Janus cassette) (2). Δ*pflB*(*spd0420*)::P_c-*erm* mutation was constructed by deletion of *pflB* except for the 5' and 3 60 bp and replacement with a P_c-*erm* cassette (P_c, constitutive promoter). Deletion insertion Δ*spd0091*::*erm*, Δ*sodA*::*erm*, Δ*tpxD*::*erm*, Δ*gpx*::*erm* and Δ*nsufU*::*erm* mutations were constructed by fusing 0 to 60 bp of 5' sequences of the targeted genes with *erm* (see Table S2). The absence of the constitutive promoter was chosen to avoid the possibility of polar effects on downstream genes. CEP::P_{mal}-*lctO* was constructed to express ectopic *lctO* at the neutral CEP site (3) as described in Table S2 to complement *lctO*::Mariner mutation. After transformation, bacteria were plated on TSAII-BA plates with antibiotics and incubated at 37°C in an atmosphere of 5% CO_2. For antibiotic selections, TSAII BA plates contained 250 µg kanamycin mL^{-1}, 150 µg spectinomycin mL^{-1}, 0.3 µg erythromycin mL^{-1}, and 250 µg streptomycin mL^{-1}. Transformants were single-colony-isolated on TSAII-BA plates containing antibiotics twice before growth in antibiotic-containing BHI broth for storage. All constructs were confirmed by PCR amplification and DNA sequencing.

Hydrogen peroxide release assays. The rate of H_2O_2 production shown in Fig. 2 was determined using the Amplex Red Hydrogen Peroxide/Peroxidase Assay kit (Invitrogen) as previously described (4). Briefly, exponential cultures were grown in BHI to OD_{620} ≈ 0.1–0.15, centrifuged briefly in a microfuge, and resuspended in fresh BHI to OD_{620} ≈ 0.06. Microtiter plates containing cells and pre-warmed reaction mixture, or a series of H_2O_2 concentration standards diluted in BHI, were incubated at 37°C in a VERSAmax tunable reader driven by SoftMax Pro software (Molecular Devices) and absorbance was read at 563 nm every 3 min for 1h. Rates of H_2O_2 production in pmol per min were calculated and normalized to the OD_{620} of the cell suspensions. Final values are shown relative to that of strain D39 (IU1690), which was included in every experiment as an internal standard.

Released hydrogen peroxide shown in Fig. 4C was measured as described previously (5, 6). Briefly, *S. pneumoniae* cultures were grown to mid-log, OD_{620} ≈0.3-0.4 at 37 °C + 5% CO_2, and harvested by centrifugation for 10 min at 10, 000 x g at 4 °C. Cells were washed with ice-cold PBS, pH 7.4 and resuspended in 5 mL PBS, pH 7.4 + 0.5 mM glucose. Cells were incubated at 37 °C + 5% CO_2 for 1 h, and harvested by centrifugation at 10, 000 x g for 10 min. The supernatant was filtered with a 0.2 µm syringe filter (Millipore), and samples stored on ice for the

measurement. Hydrogen peroxide levels were measured using the Pierce Quantitative Peroxide assay kit using a standard curve from 1 μM to 200 μM H_2O_2.

H_2O_2 sensitivity assay. Hydrogen peroxide sensitivity assays were performed as described (4) except that survival was calculated after 5, 10 and 15 minutes of treatment and the serial dilutions were done in 1x PBS.

SUPPLEMENTARY REFERENCES

1. **Tsui HC, Mukherjee D, Ray VA, Sham LT, Feig AL, Winkler ME.** 2010. Identification and characterization of noncoding small RNAs in *Streptococcus pneumoniae* serotype 2 strain D39. J Bacteriol **192:**264-279.
2. **Sung CK, Li H, Claverys JP, Morrison DA.** 2001. An *rpsL* cassette, Janus, for gene replacement through negative selection in *Streptococcus pneumoniae*. Appl Environ Microbiol **67:**5190-5196.
3. **Guiral S, Henard V, Laaberki MH, Granadel C, Prudhomme M, Martin B, Claverys JP.** 2006. Construction and evaluation of a chromosomal expression platform (CEP) for ectopic, maltose-driven gene expression in *Streptococcus pneumoniae.* Microbiology **152:**343-349.
4. **Ramos-Montanez S, Tsui HC, Wayne KJ, Morris JL, Peters LE, Zhang F, Kazmierczak KM, Sham LT, Winkler ME.** 2008. Polymorphism and regulation of the *spxB* (pyruvate oxidase) virulence factor gene by a CBS-HotDog domain protein (SpxR) in serotype 2 *Streptococcus pneumoniae*. Mol Microbiol **67:**729-746.
5. **Pericone CD, Overweg K, Hermans PW, Weiser JN.** 2000. Inhibitory and bactericidal effects of hydrogen peroxide production by *Streptococcus pneumoniae* on other inhabitants of the upper respiratory tract. Infect Immun **68:**3990-3997.
6. **Hajaj B, Yesilkaya H, Benisty R, David M, Andrew PW, Porat N.** 2012. Thiol peroxidase is an important component of *Streptococcus pneumoniae* in oxygenated environments. Infect Immun **80:**4333-4343.
